# Supplementary material for: Interplay of Histone Marks with Serine ADP-Ribosylation
Source: Cell Rep. 2018 Sep 25;24(13):3488–3502.e5. doi: 10.1016/j.celrep.2018.08.092 (PMC6172693; doi:10.1016/j.celrep.2018.08.092)
Supplement: Document S1. Figures S1–S6 [file mmc1.pdf]

Cell Reports, Volume *24*

Supplemental Information

Interplay of Histone Marks with Serine ADP-Ribosylation

Edward Bartlett, Juan José Bonfiglio, Evgeniia Prokhorova, Thomas Colby, Florian Zobel, Ivan Ahel, and Ivan Matic

Figure S1

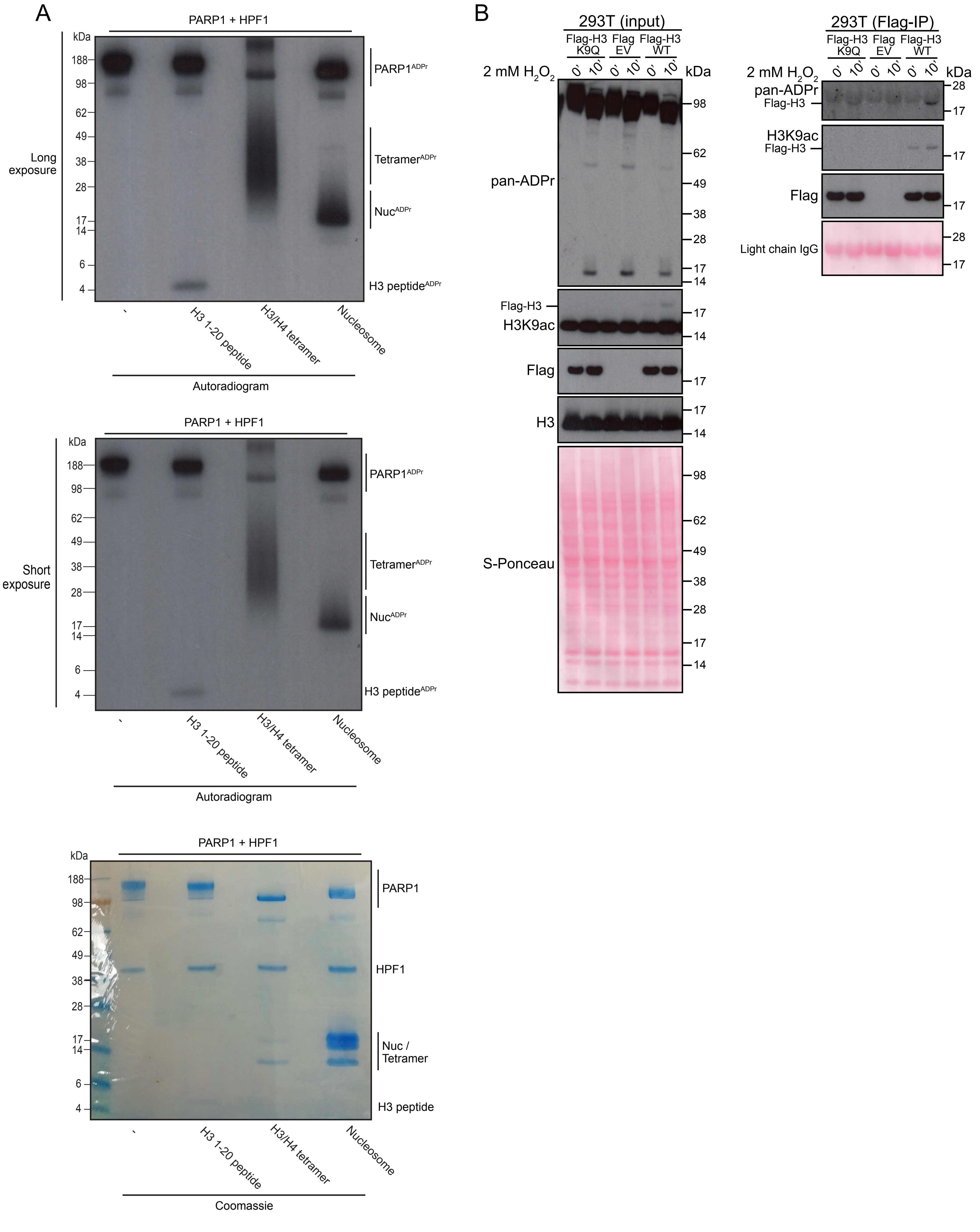
A
PARP1 + HPF1
kDa
188
98
62
49
38
28
17
14
6
4
Long exposure
PARP1^ADPr
Tetramer^ADPr
Nuc^ADPr
H3 peptide^ADPr
-
H3 1-20 peptide
H3/H4 tetramer
Nucleosome
Autoradiogram
PARP1 + HPF1
Short exposure
PARP1^ADPr
Tetramer^ADPr
Nuc^ADPr
H3 peptide^ADPr
Autoradiogram
PARP1 + HPF1
PARP1
HPF1
Nuc / Tetramer
H3 peptide
Coomassie
B
293T (input)
Flag-H3 K9Q
Flag EV
Flag-H3 WT
2 mM H2O2
0'
10'
kDa
pan-ADPr
Flag-H3
H3K9ac
Flag
H3
S-Ponceau
293T (Flag-IP)
Light chain IgG

Figure S1. Related to Figure 1

(A) Autoradiogram showing PARP1/HPF1 mediated ADPr of H3 (1-20aa) peptide, H3/H4 tetramer and recombinant nucleosome. Coomassie staining of the SDS-PAGE is included.

(B) H3K9Q mutant (acetylation mimetic) prevents Ser-ADPr of histone H3 following DNA damage. 293T wt cells were transfected with the same amount of EV or plasmid expressing WT or K9Q Flag-tagged histone H3 protein, and treated for 10 minutes with H_2O_2.

Figure S2

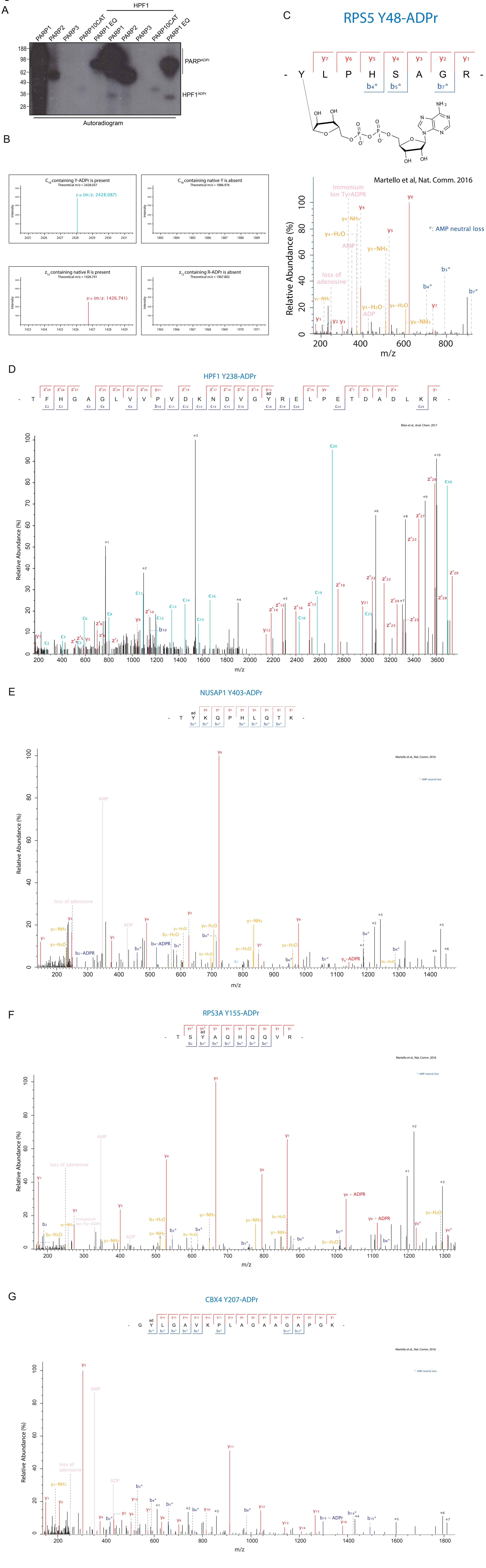

A
HPF1
PARP1
PARP2
PARP3
PARP10CAT
PARP1 EQ
PARP^{ADPr}
HPF1^{ADPr}
Autoradiogram
B
C_{18} containing Y-ADPr is present
Theoretical m/z = 2428.037
c_{18} (m/z: 2428.087)
C_{18} containing native Y is absent
Theoretical m/z = 1886.976
z_{12} containing native R is present
Theoretical m/z = 1426.741
z_{12} (m/z: 1426.741)
z_{12} containing R-ADPr is absent
Theoretical m/z = 1967.802
Intensity
C
RPS5 Y48-ADPr
Martello et al, Nat. Comm. 2016
Immonium Ion TyrADPR
loss of adenosine
AMP
*: AMP neutral loss
Relative Abundance (%)
m/z
D
HPF1 Y238-ADPr
Bilan et al., Anal. Chem. 2017
Relative Abundance (%)
m/z
E
NUSAP1 Y403-ADPr
Martello et al., Nat. Comm. 2016
*: AMP neutral loss
AMP
loss of adenosine
ADP
Relative Abundance (%)
m/z
F
RPS3A Y155-ADPr
Martello et al., Nat. Comm. 2016
*: AMP neutral loss
AMP
loss of adenosine
Immonium Ion Tyr-ADPr
ADP
Relative Abundance (%)
m/z
G
CBX4 Y207-ADPr
Martello et al., Nat. Comm. 2016
*: AMP neutral loss
AMP
loss of adenosine
ADP
Relative Abundance (%)
m/z

Figure S2. Discovery of Tyrosine as a target residue for ADPr. Related to Figure 2

(A) A longer exposure of the autoradiogram from Figure 2D, showing a panel of PARPs incubated with HPF1 protein, with visible PARP1 EQ mediated HPF1 ADPr.

(B) Zoom of the spectrum of Figure 2B in different m/z ranges showing the presence of sequence ions that pinpoint ADPr to Y238 (left panels), and the absence of signal in the region that would correspond to sequence ions containing R239-ADPr (right panels).

(C) High-resolution HCD fragmentation spectrum of an RPS5 peptide modified by ADP-ribose obtained from reprocessing a published high-quality proteomics dataset (Martello et al., 2016). Note that the presence of a Tyr-ADPr diagnostic peak in this HCD spectrum (Immonium Ion TyrADPR on the figure) enhances the confidence to assign tyrosine 48 as the modified residue.
* AMP neutral loss. The immonium ion for ADPr-Tyrosine has also undergone the loss of AMP.

(D) High-resolution ETD fragmentation spectrum of a HPF1 peptide modified by ADP-ribose on tyrosine 238 from reprocessing a published high-quality proteomics dataset (Bilan et al., 2017). *[1] peaks corresponding to co-isolated species in their original charge state. Multiple species in charge state 2-5 passed through the quadruopoley and could not be completely deconvoluted. *[2] unassigned peak (m/z = 1098.539). *[3] peak corresponding to an unfragmented co-eluting, co-isolated +2 precursor deconvoluted into the +1 state. *[4] unassigned peak (m/z = 1905.373). *[5] peak corresponding to an unfragmented co-eluting, co-isolated +3 precursor deconvoluted into the +1 state. *[6] peak corresponding to an unfragmented co-eluting, co-isolated +4 precursor deconvoluted into the +1 state. *[7] peak corresponding to the identified +5 precursor after a complete loss of ADPr due to HCD fragmentation*. *[8] peak corresponding to the Z28 ion of the identified +5 precursor after a loss of Adenosine due to HCD fragmentation[a]. *[9] peak corresponding to the identified +5 precursor after a loss of ADP due to HCD fragmentation[a]. *[10] peak corresponding to the identified +5 precursor after a loss of Adenosine due to HCD fragmentation[a].
[a]These features illustrate some disadvantage of supplemental HCD activation. The assigned products illustrate that HCD-induced fragmentation is taking place on the identified modified 5+ peptide and its ETD fragments, thus complicating the spectrum.

(E) High-resolution HCD fragmentation spectra of a NUSAP peptide modified by Tyr-ADP-ribose obtained from reprocessing a published high-quality proteomics dataset (Martello et al., 2016). *[1] peak corresponding to an unfragmented co-eluting, co-isolated +2 precursor deconvoluted into the +1 state. *[2] peak corresponding to the identified precursor after a loss of ADPr and H2O. *[3] peak corresponding to the identified precursor after a loss of ADPr. *[4] peak corresponding to the identified precursor after a loss of AMP and H2O. *[5] peak corresponding to the identified precursor after a loss of AMP. *[6] peak corresponding to the identified precursor after a loss of AMP and an addition of H2O.

(F) High-resolution HCD fragmentation spectra of a RPS3A peptide modified by Tyr-ADP-ribose obtained from reprocessing a published high-quality proteomics dataset (Martello et al., 2016). *[1] peak corresponding to the identified precursor after a loss of ADPr and H2O. *[2] peak corresponding to the identified precursor after a loss of ADPr. *[3] peak corresponding to the identified precursor after a loss of AMP.

(G) High-resolution HCD fragmentation spectra of a CBX4 peptide modified by Tyr-ADP-ribose obtained from reprocessing a published high-quality proteomics dataset (Martello et al., 2016). *[1] peak corresponding to an internal fragment of the precursor (_PLAGAAGA_). *[2] peak corresponding to an internal fragment of the precursor (_KPLAGAAGA_). *[3] peak corresponding to an internal fragment of the precursor (_GAVKPLAGAA_). *[4] peak corresponding to an unfragmented co-eluting, co-isolated +2 precursor deconvoluted into the +1 state. *[5] peak corresponding to the identified precursor after a loss of ADPr. *[6] peak corresponding to the identified precursor after a loss of AMP. *[7] peak corresponding to the identified precursor after a loss of AMP and H2O.

Figure S3

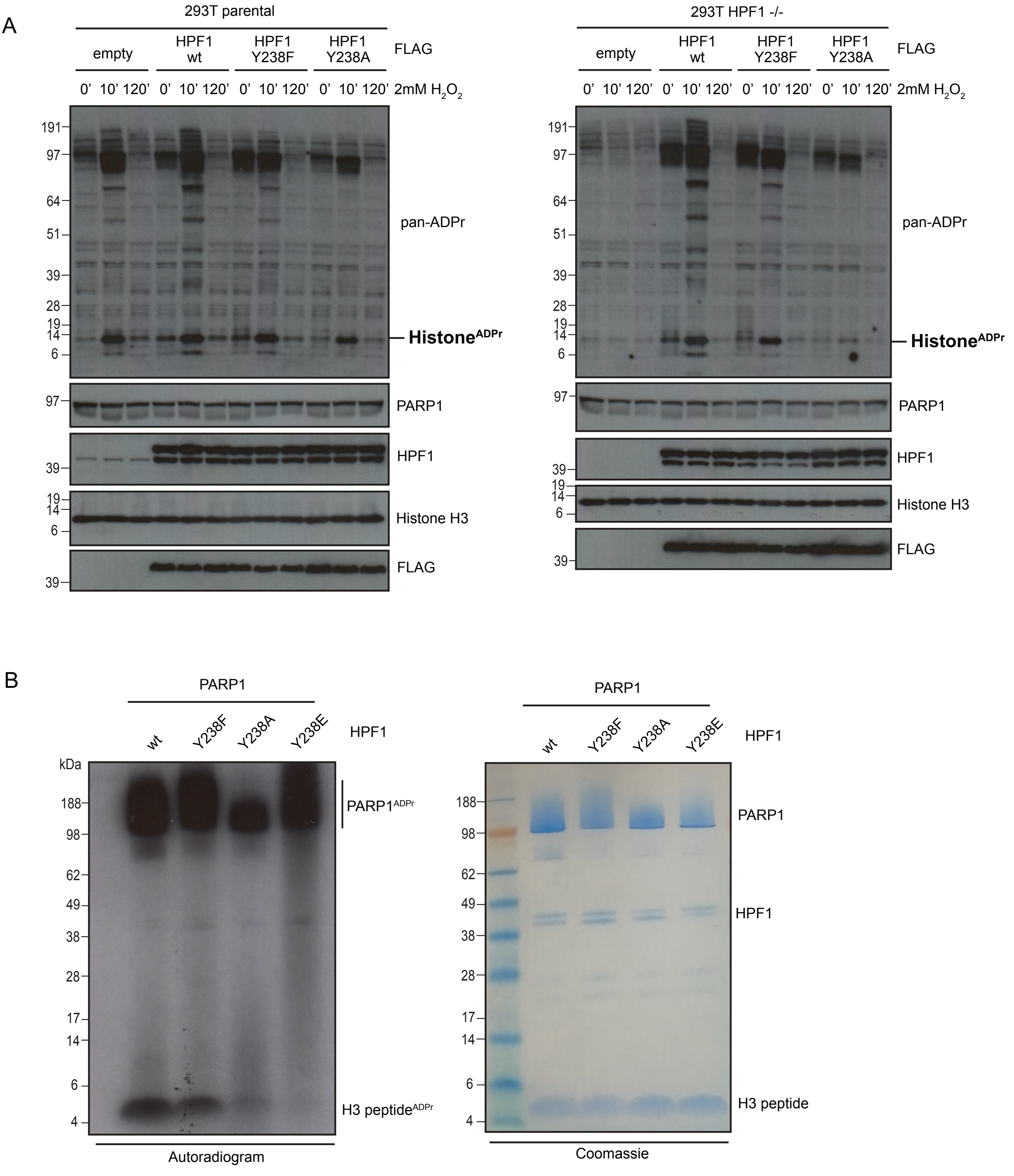

Figure S3. Mutation of HPF1 Tyrosine 238 to phenylalanine supports global Ser-ADP-ribosylation. Related to Figure 2

(A) Complementation of HPF1 in wt and -/- human cells. 293T wt or HPF1 -/- cells were transfected with the same amount of empty vector or plasmid expressing WT, Y238F or Y238A Flag-tagged HPF1 protein, and treated for 0, 10 or 120 minutes with H2O2.

(B) Autoradiogram showing ADPr of H3 peptide following incubation with PARP1 and either HPF1 wt, Y238F, Y238A or Y238E proteins. Coomassie staining of the SDS-PAGE is included.

Figure S4

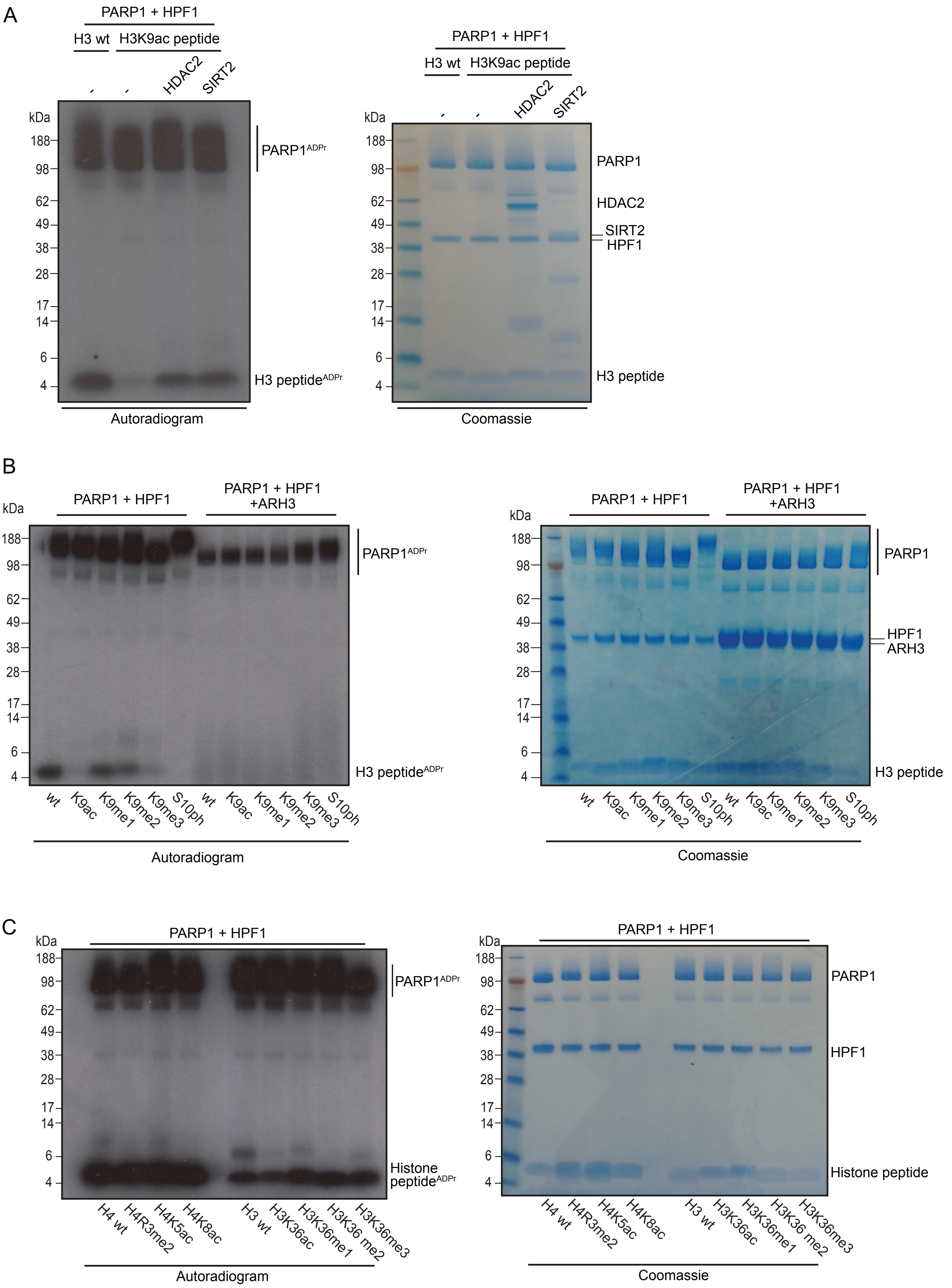

A
PARP1 + HPF1
H3 wt
H3K9ac peptide
HDAC2
SIRT2
kDa
188
98
62
49
38
28
17
14
6
4
PARP1ADPr
H3 peptideADPr
Autoradiogram
PARP1
HDAC2
SIRT2
HPF1
H3 peptide
Coomassie
B
PARP1 + HPF1
PARP1 + HPF1 +ARH3
wt
K9ac
K9me1
K9me2
K9me3
S10ph
ARH3
C
Histone peptideADPr
Histone peptide
H4 wt
H4R3me2
H4K5ac
H4K8ac
H3 wt
H3K36ac
H3K36me1
H3K36 me2
H3K36me3

Figure S4. Canonical H3 histone marks reduce the efficiency of H3S10ADPr on H3 peptide. Related to Figure 3

(A) Autoradiogram showing PARP1/HPF1 ADPr signal on H3K9ac peptide following prior deacetylation treatments with HDAC2 and SIRT2. Coomassie staining of the SDS-PAGE is included.

(B) Autoradiogram showing ADPr, with or without subsequent addition of ARH3, with H3 (1-20aa) WT, K9ac, K9me1, K9me2, K9me3 and S10ph peptides. Coomassie staining of the SDS-PAGE is included.

(C) As in panel (B) except with H4 (1-23) WT, R3me2, K5ac, K8ac, H2A (1-17aa) WT, H3 (27-45aa) WT, K36ac, K36me1, K36me2, K36me3 peptides. Coomassie staining of the SDS-PAGE is included.

Figure S5

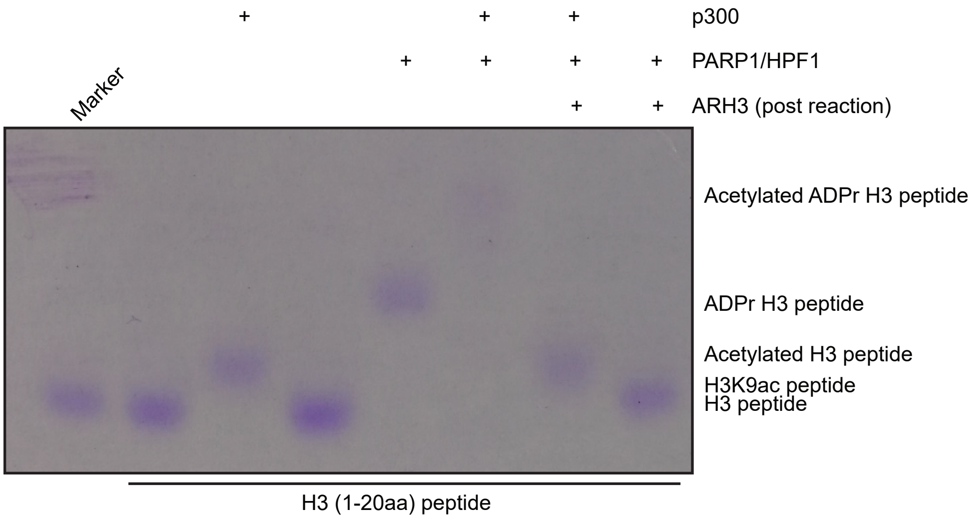

Figure S5. H3S10ADPr reduces the efficiency of subsequent H3K9 acetylation. Related to Figure 4 and 5

TBE gel of PARP1/HPF1 and/or p300 treatments of H3 (1-20aa) peptide. For details of the peptide separate technique please refer to Figure 5 and the related text. H3K9ac peptide was used as a marker. H3 peptides were ADP-ribosylated by PARP1/HPF1, prior to p300 acetylation treatment. One reaction (lane 7) was stopped after p300 incubation of Ser-ADP-ribosylated H3 peptide, then supplemented with ARH3 to remove ADPr (please note the mobility shift after the ARH3 treatment). ARH3 glycosylhydrolase removal of Ser-ADPr from non-acetylated H3 peptide is included as an additional control. Given the almost complete lack of H3K9ac detection by antibody in lane 6 of Figure 4A and a small but significant band shift in this figure (compare lane 7 to lanes 3 and 8), we note that Ser-ADPr H3 peptide can be acetylated by p300 at additional distal lysine sites in addition to K9.

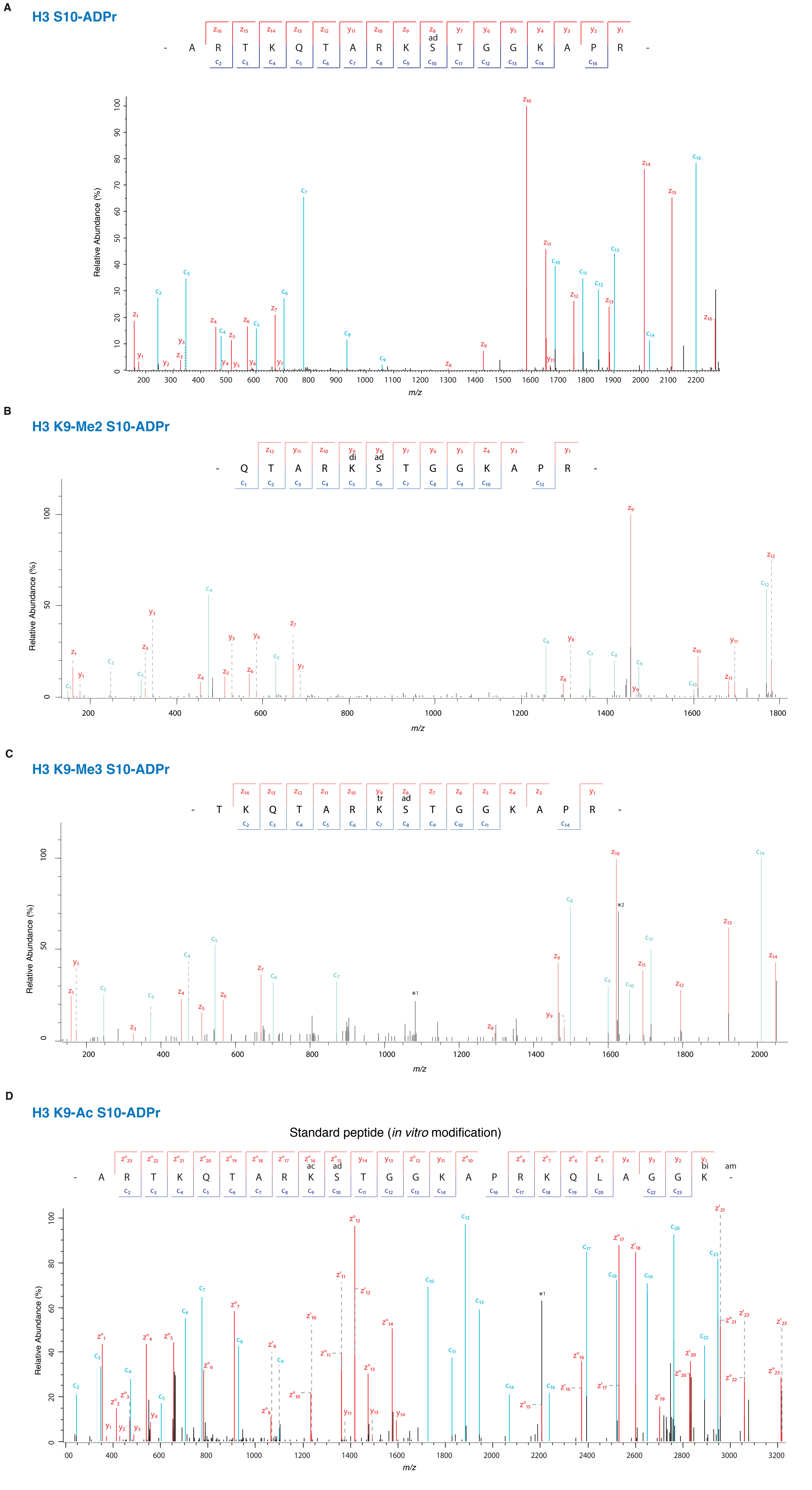
A
H3 S10-ADPr
- A R T K Q T A R K S T G G K A P R -
ad
Relative Abundance (%)
m/z
B
H3 K9-Me2 S10-ADPr
- Q T A R K S T G G K A P R -
di
ad
Relative Abundance (%)
m/z
C
H3 K9-Me3 S10-ADPr
- T K Q T A R K S T G G K A P R -
tr
ad
Relative Abundance (%)
m/z
D
H3 K9-Ac S10-ADPr
Standard peptide (in vitro modification)
- A R T K Q T A R K S T G G K A P R K Q L A G G K -
ac
ad
bi
am
Relative Abundance (%)
m/z

Figure S6. H3S10ADPr can coexist with mono-, di-, and tri-methylation of H3K9. Related to Figure 6

(A) High-resolution ETD fragmentation spectrum of a H3 peptide modified by ADP-ribose on serine 10 obtained from (Leidecker et al., 2016).

(B) High-resolution ETD fragmentation spectrum of a H3 peptide modified by di-methyl on lysine 9 and ADP-ribose on serine 10 obtained from (Leidecker et al., 2016).

(C) High-resolution ETD fragmentation spectrum of a H3 peptide modified by tri-methyl on lysine 9 and ADP-ribose on serine 10 obtained from (Leidecker et al., 2016). $*^1$ peak corresponding to an unfragmented co-eluting, co-isolated +2 precursor deconvoluted into the +1 state. $*^2$ peak corresponding to an unfragmented co-eluting, co-isolated +3 precursor deconvoluted into the +1 state.

(D) High-resolution ETD fragmentation spectrum of a H3-K9AcS10ad standard peptide (*in vitro* modification). $*^1$ peak corresponding to an unfragmented co-eluting, co-isolated +4 precursor deconvoluted into the +1 state.
