# Supplementary material for: Interplay of Histone Marks with Serine ADP-Ribosylation
Source: Cell Rep. 2018 Sep 25;24(13):3488–3502.e5. doi: 10.1016/j.celrep.2018.08.092 (PMC6172693; doi:10.1016/j.celrep.2018.08.092)
Supplement: Document S2. Article plus Supplemental Information [file mmc2.pdf]

Article

Cell Reports

Interplay of Histone Marks with Serine ADP-Ribosylation

Graphical Abstract

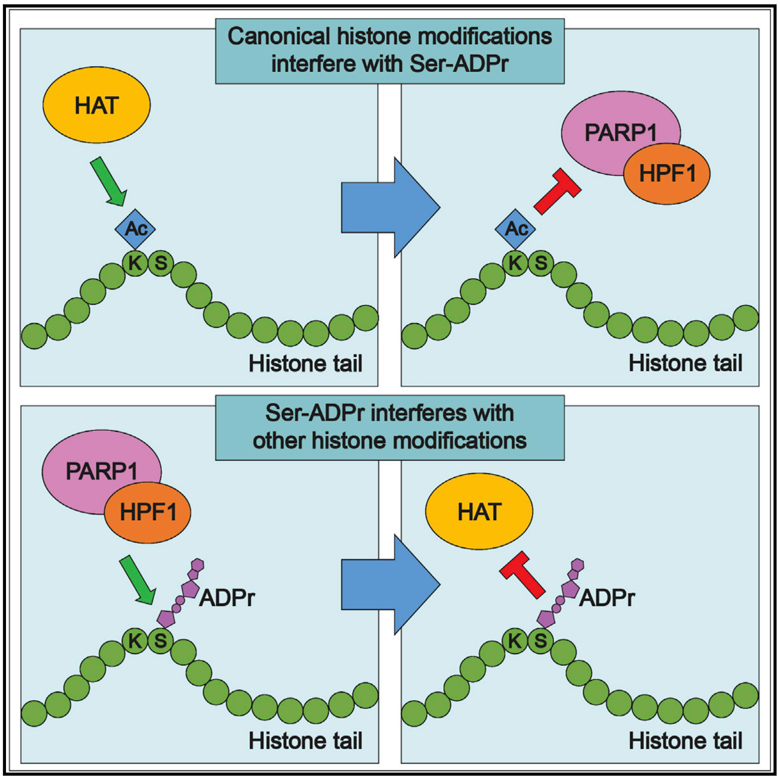

Highlights

- Histone serine ADP-ribosylation and proximal acetylation are mutually exclusive
- PARP inhibitors block the DNA damage-induced histone H3 deacetylation
- Tyrosine residues are ADP-ribosylation targets
- Inverted polarity electrophoresis simplifies the study of histone marks

Authors

Edward Bartlett, Juan José Bonfiglio, Evgeniia Prokhorova, Thomas Colby, Florian Zobel, Ivan Ahel, Ivan Matic

Correspondence

ivan.ahel@path.ox.ac.uk (I.A.),
imatic@age.mpg.de (I.M.)

In Brief

Bartlett et al. demonstrate that serine ADP-ribosylation and proximal phosphorylation and acetylation are mutually exclusive at the N-terminal tails of core histones. They also expand the repertoire of ADP-ribosylation target residues by providing evidence for tyrosine ADP-ribosylation on HPF1 and several other proteins.

Bartlett et al., 2018, Cell Reports *24*, 3488–3502
September 25, 2018 © 2018 The Author(s).
https://doi.org/10.1016/j.celrep.2018.08.092

CellPress

OPEN ACCESS
CellPress

Cell Reports
Article

Interplay of Histone Marks with Serine ADP-Ribosylation

Edward Bartlett,[1,3] Juan José Bonfiglio,[2,3] Evgeniia Prokhorova,[1] Thomas Colby,[2] Florian Zobel,[1] Ivan Ahel,[1,4,*] and Ivan Matic[2,*]
[1]Sir William Dunn School of Pathology, University of Oxford, South Parks Road, Oxford OX1 3RE, UK
[2]Max Planck Institute for Biology of Ageing, Joseph-Stelzmann-Strasse 9b, Cologne 50931, Germany
[3]These authors contributed equally
[4]Lead Contact
*Correspondence: ivan.ahel@path.ox.ac.uk (I.A.), imatic@age.mpg.de (I.M.)
https://doi.org/10.1016/j.celrep.2018.08.092

SUMMARY

Serine ADP-ribosylation (Ser-ADPr) is a recently discovered protein modification that is catalyzed by PARP1 and PARP2 when in complex with the eponymous histone PARylation factor 1 (HPF1). In addition to numerous other targets, core histone tails are primary acceptors of Ser-ADPr in the DNA damage response. Here, we show that specific canonical histone marks interfere with Ser-ADPr of neighboring residues and vice versa. Most notably, acetylation, but not methylation of H3K9, is mutually exclusive with ADPr of H3S10 *in vitro* and *in vivo*. We also broaden the *O*-linked ADPr spectrum by providing evidence for tyrosine ADPr on HPF1 and other proteins. Finally, we facilitate wider investigations into the interplay of histone marks with Ser-ADPr by introducing a simple approach for profiling posttranslationally modified peptides. Our findings implicate Ser-ADPr as a dynamic addition to the complex interplay of modifications that shape the histone code.

INTRODUCTION

ADP-ribosylation (ADPr) is a clinically important posttranslational modification (PTM) that controls many cellular processes, including DNA repair, transcription, translation, and chromatin remodeling (Gupte et al., 2017; Posavec Marjanović et al., 2017; Palazzo et al., 2017; Cohen and Chang, 2018). The ADPr reaction consists of the enzymatic transfer of ADPr from positively charged nicotinamide adenine dinucleotide (NAD^+) onto an acceptor molecule with the simultaneous release of nicotinamide (Gupte et al., 2017; Pascal and Ellenberger, 2015). Poly(ADPr) polymerases (PARPs) are the major family of enzymes that perform ADPr, and 17 PARP family members are encoded in the human genome (Barkauskaite et al., 2015). PARP1 and PARP2 are the most studied members of the family and are particularly known for their key roles in the DNA damage response (DDR) (Martin-Hernandez et al., 2017; Pascal and Ellenberger, 2015).

PARPs modify proteins at specific residues, and several amino acids, most commonly glutamate (Glu) and aspartate (Asp) but also arginine (Arg), lysine (Lys), and cysteine (Cys), have been reported to be ADPr (Vyas et al., 2014; Vivelo and Leung, 2015; Crawford et al., 2018). Recently, we identified serine ADPr (Ser-ADPr) as an elusive type of histone PTMs that target specific Ser residues (Leidecker et al., 2016) and revealed the basic molecular mechanisms underlying Ser-ADPr conjugation and its reversal. Specifically, we established Ser as a target of PARP1/2-mediated ADPr (Bonfiglio et al., 2017b) and described histone PARylation factor 1 (HPF1/C4orf27) as the PARP1/2-interacting protein (Gibbs-Seymour et al., 2016) required for conferring specificity toward Ser (Bonfiglio et al., 2017b). We also characterized ADPr 3 (ARH3, or ADPRHL2) as the hydrolase responsible for Ser-ADPr removal (Fontana et al., 2017). Further studies identified hundreds of DNA damage-induced Ser-ADPr sites in proteins involved in DNA repair, transcription, and chromatin organization (Bonfiglio et al., 2017b; Abplanalp et al., 2017) and revealed that Ser-ADPr is the major type of ADPr in the regulation of the DDR (Palazzo et al., 2018).

Ser-ADPr core histone marks are localized on N-terminal tails (Leidecker et al., 2016), which are heavily decorated with a plethora of dynamic, covalent modifications, including phosphorylation, acetylation, methylation, and ubiquitylation (Huang et al., 2015). Specific combinations of these marks act together to regulate a host of important nuclear functions, such as chromatin compaction and dynamics, transcription, replication, and DNA repair (Lawrence et al., 2016; Tan et al., 2011; Huang et al., 2015). Many studies have already been conducted on various histone modifications, yet all of them have overlooked Ser-ADPr because this PTM remained elusive until recently (Leidecker et al., 2016). Conversely, despite their focus on histones, studies centered on Ser-ADPr have so far investigated this PTM independent of other histone marks (Leidecker et al., 2016; Bonfiglio et al., 2017b; Fontana et al., 2017; Bilan et al., 2017).

In this paper, we provide insights into the interplay between Ser-ADPr and canonical histone marks. Furthermore, by characterizing the PARP/HPF1-catalyzed ADPr consensus motif, we determine the relative significance of the preceding basic residue and discover tyrosine as an acceptor for ADPr. The resulting interplay analysis examines the effect of surrounding histone PTMs and shows that certain specific acetylation and phosphorylation marks can inhibit Ser-ADPr and vice versa. To broaden and improve studies of histone marks interplay, we introduce a method for visualization of modified as well as unmodified counterpart peptides.

 © 2018 The Author(s).
This is an open access article under the CC BY license (http://creativecommons.org/licenses/by/4.0/).

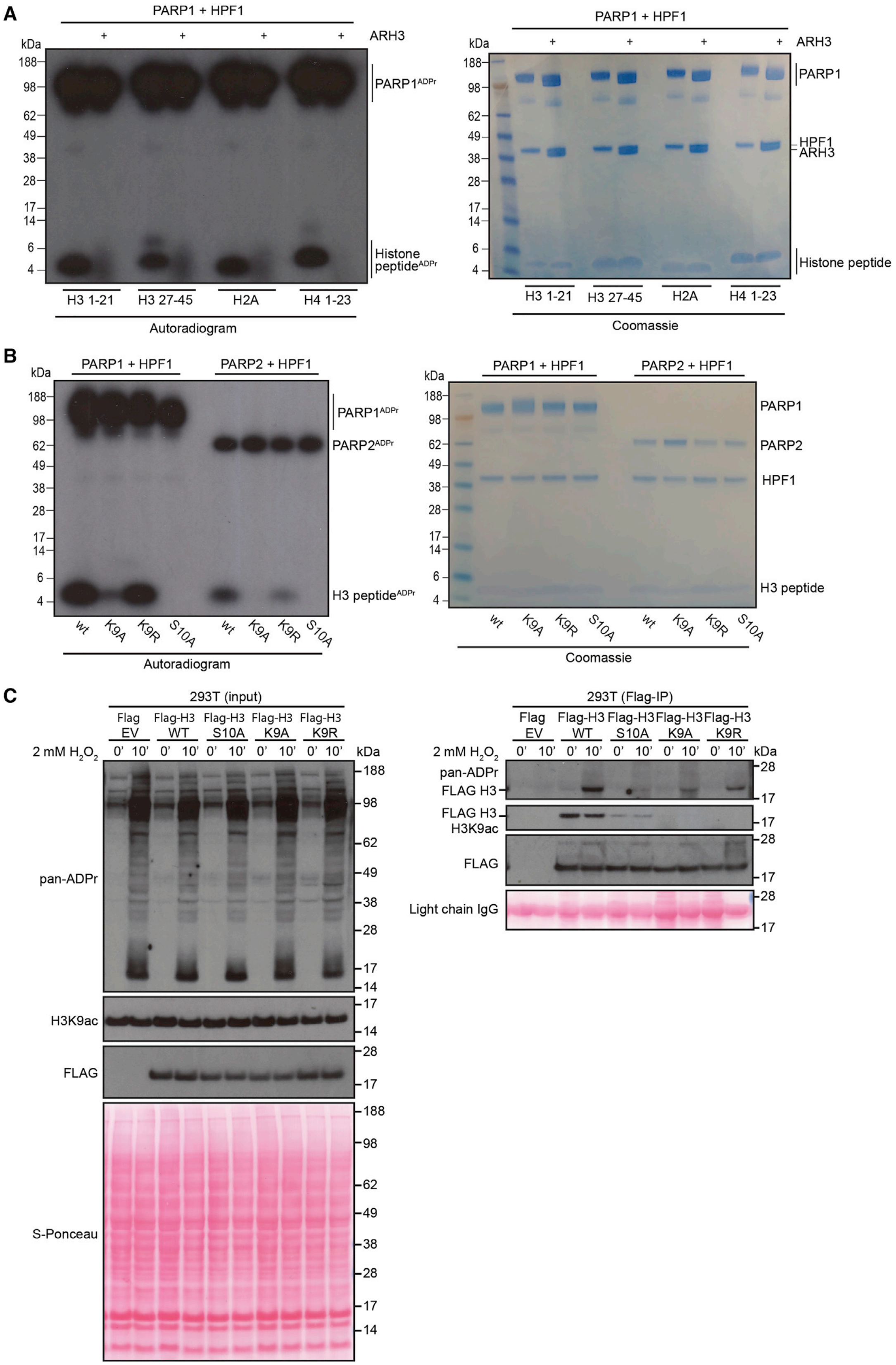

(legend on next page)

RESULTS

Factors Influencing Ser-ADPr of Histone Peptides

To analyze the substrate properties that influence Ser-ADPr, we investigated sequence features that may affect the efficiency of *in vitro* histone peptide ADPr reactions. Our previous proteomics data provided a short consensus motif for *in vivo* Ser-ADPr with either Lys or Arg N-terminal to the target Ser (Leidecker et al., 2016; Bonfiglio et al., 2017b). Based on these observations, we incubated PARP1 and HPF1 with a variety of histone peptides, each containing an Lys-Ser (KS) motif known to be the modification site *in vivo* (Leidecker et al., 2016). Similar to what we reported before (Bonfiglio et al., 2017b), we observed that two different histone H3 peptides as well as H2A and H4 peptides were modified by the HPF1/PARP1 complex *in vitro* (Figure 1A). The Ser-ADPr glycosylhydrolase ARH3 (Fontana et al., 2017) was able to efficiently remove the ADP-ribose on all of the analyzed peptides (Figure 1A). We also compared the efficiency of H3 peptide 1–20 modification to that of the H3/H4 tetramer and the whole nucleosome. As shown in Figure S1A, peptide modification is not dramatically lower, especially considering the additional ADPr sites on the histone proteins and that this H3 peptide is mostly mono-ADPr *in vitro* (Bonfiglio et al., 2017b). These experiments establish that KS motifs in a variety of histone peptides can be modified efficiently and reversibly, demonstrating the utility of the histone peptide as a tractable *in vitro* assay for histone Ser-ADPr.

Next, we opted to focus on H3 Ser10 (H3S10) ADPr, because this site was previously shown to be the primary ADPr site on H3 *in vivo* (Palazzo et al., 2018). We investigated how alterations of the key KS residues affect the modification profile of the H3 histone peptide *in vitro*. Based on our previous finding that both PARP1 and PARP2 can modify this H3 peptide in the presence of HPF1 (Bonfiglio et al., 2017b), we examined both PARPs with variations on the KS motif. Substitution of Ser10 with alanine (Ala) led to a complete loss of the modification (Figure 1B), as we have previously shown (Bonfiglio et al., 2017b). Changing the neighboring Lys residue into Arg or Ala had varying effects on histone Ser-ADPr. The H3 peptide containing the K9R mutation was still modified, albeit to a lesser extent than wild-type (WT) peptide. In contrast, the H3K9A mutation strongly (but not completely) inhibited histone H3 Ser-ADPr (Figure 1B), highlighting the importance of a basic residue preceding the Ser. Both PARP1 and PARP2 modified the peptide panel with similar profiles, although PARP1 catalyzed the reactions more efficiently under the conditions used.

We further confirmed the importance of the consensus KS motif for Ser-ADPr *in vivo*. We transfected 293T cells with FLAG-tagged histone H3 WT, K9A, K9R, K9Q, or S10A mutant H3 and assessed ADPr efficiency, as described previously (Palazzo et al., 2018). DNA damage was induced by the treatment with 2 mM hydrogen peroxide (H_2O_2), followed by FLAG-immunoprecipitation (FLAG-IP). Western blotting was performed using a pan-ADPr reagent that recognizes all forms of cellular ADPr (Figures 1C and S1B). The ADPr patterns obtained were similar to those observed in our *in vitro* reactions. To note, by using a specific anti-H3K9ac antibody, we show that the KS motif is also important for K9 acetylation *in vivo* (Figure 1C, FLAG-IP).

These data extend our previous findings that the KS and RS motifs are preferred targets for Ser-ADPr and exclude the possibility that Lys rather than Ser is the modification target.

Discovery of Tyrosine as a Target Residue for ADPr

ADPr of Ser led us to question whether a hydroxyl group is sufficient and necessary to target an amino acid for ADPr when adjacent to Lys. We therefore decided to substitute H3S10 with threonine (Thr) and tyrosine (Tyr), the two other residues that contain hydroxyl groups, and additionally Glu and Asp as further controls. Not only were we unable to detect ADPr on Glu and Asp but also on Thr residues (Figure 2A). This suggests that although chemically similar to Ser, the additional methyl group on Thr interferes with the ADPr reaction mediated by PARP1/HPF1. In fact, in none of our previous proteomic analyses (Leidecker et al., 2016; Bonfiglio et al., 2017b) were we able to detect Thr-ADPr. Conversely, we identified a reproducible modification of Tyr when we introduced this amino acid instead of Ser10 (Figure 2A). Because Tyr has not previously been described as a substrate for ADPr, we sought mass spectrometric evidence for Tyr-ADPr. Although we could not detect Tyr-ADPr in our histone proteomics data (Leidecker et al., 2016), we confidently identified Tyr-ADPr of HPF1 in an *in vitro* reaction containing PARP1 (Figures 2B and S2B). We could also identify Ser97 in HPF1 as another site modified in this reaction (Figure 2C). These data suggested that PARP1 was the enzyme responsible for HPF1 Tyr-ADPr modification. To follow up on this point, we modified recombinant HPF1 using a panel of different PARPs and radioactively labeled NAD. We could observe a low but reproducible modification by PARP1 and possibly by PARP2 (Figures 2D, S2A, and S2E). This modification is at least partly dependent on the assembly of the PARP1/HPF1 complex, because the modification of the HPF1 R239A mutant protein (previously shown to be deficient in interacting with PARP; see Gibbs-Seymour et al., 2016) was significantly reduced (Figure 2E).

To confirm the ADPr of HPF1 *in vivo*, we overexpressed and immunoprecipitated FLAG-tagged HPF1 WT, S97A, and Y238A mutant proteins from 293T cells, as was described above for histone H3. We observed that HPF1 is significantly modified in cells even in undamaged conditions (Figure 2F). We did not detect a major effect of the S97A mutation on the modification

Figure 1. Modifiers of Serine-ADP-Ribosylation of Histone Peptides

(A) Autoradiogram showing ADPr, and subsequent ARH3-mediated glycohydrolysis of H3 1–20aa, H3 27–45aa, H2A 1–17aa, and H4 1–23aa peptides. Coomassie staining of the SDS-PAGE is included and represents the loading control.

(B) Autoradiogram showing PARP1/2 + HPF1-mediated ADPr of H3 peptide with Lys9 substituted by Ala and Arg, and Ser10 substituted by Ala. Coomassie staining of the SDS-PAGE is included.

(C) 293T cells were transfected with the same amount of empty vector (EV) or plasmid expressing WT, K9A, K9R, or S10A FLAG-tagged histone H3 protein and treated for 10 min with H_2O_2. Inputs (A) and FLAG-IPs (B) were analyzed by western blotting.

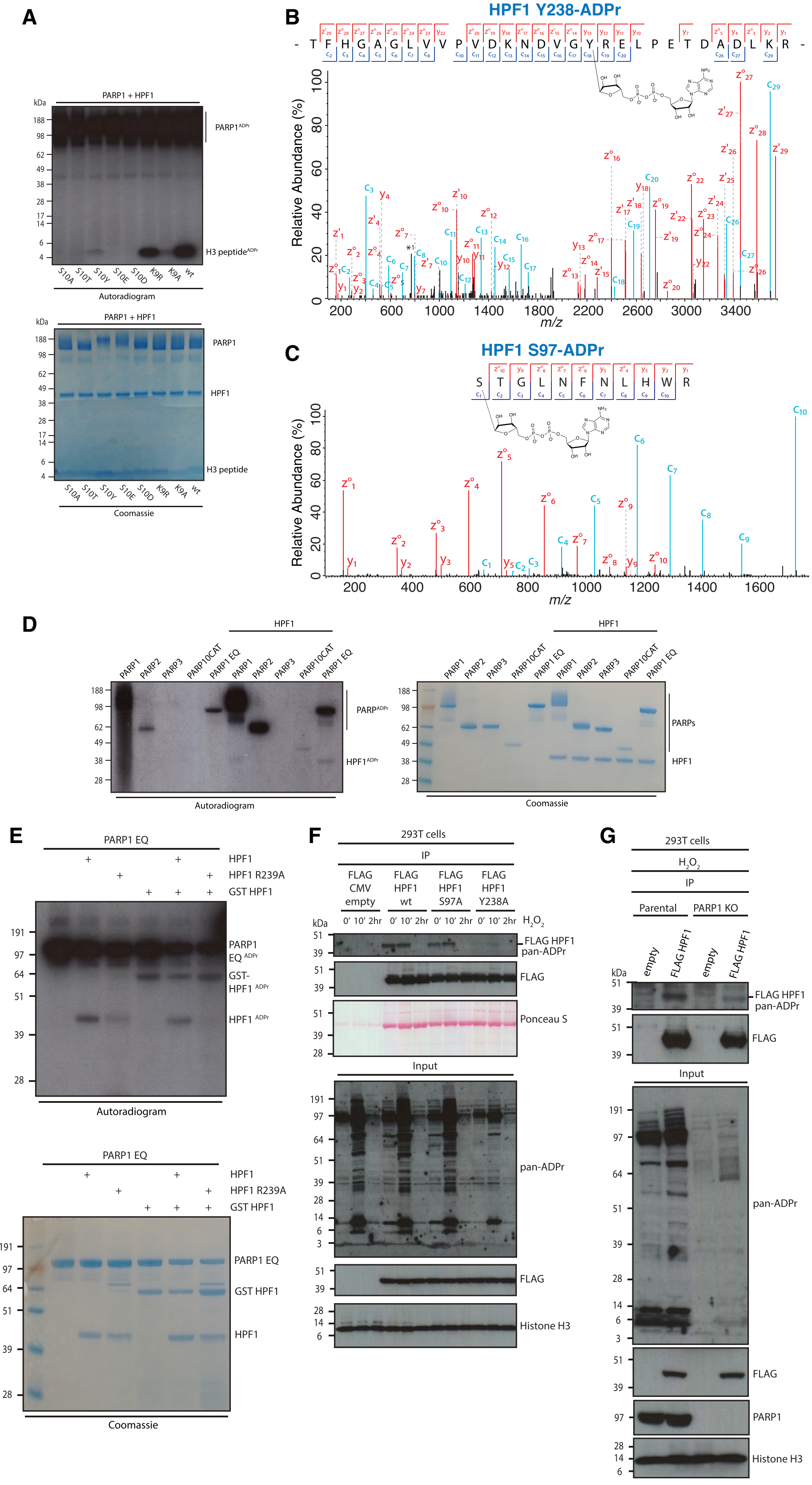

(legend on next page)

of HPF1. However, mutation of the Tyr238 site to Ala had a profound effect on the HPF1 ADPr signal (Figure 2F). This defect may be at least partly due to a reduced ability of the Y238A mutant to interact with PARP1 and to stimulate ADPr (Gibbs-Seymour et al., 2016). To further prove that HPF1 ADPr is dependent on PARP1, we performed FLAG-IP in PARP1 knockout (KO) 293T cells. As can be seen in Figure 2G, HPF1 ADPr is largely missing in PARP1 KO cells. It is likely that the remaining HPF1 modification is due to PARP2 activity. ADPribosylation of Tyr238 is not essential for the global HPF1-dependent ADPr of histones because the non-modifiable Y238F HPF1 mutant supports this activity both in cells and *in vitro* (Figures S3A and S3B).

While it appears that there may be multiple ADPr sites on HPF1, we were able to confirm the ADPr of Y238 on HPF1 in cell extracts by ADPr mapping through reprocessing (Matic et al., 2012) of a published dataset (Bilan et al., 2017) (Figure S2D). Reanalysis of a large-scale ADPr dataset (Martello et al., 2016) revealed four additional high-certainty Tyr-ADPr target proteins (Figures S2C and S2E–S2G). Although the type of mass spectrometric analysis used to generate the latter dataset is suboptimal (for additional information about the inadequacies of the higher-energy collisional dissociation [HCD] technology for ADPr site mapping, please refer to Bonfiglio et al., 2017a), our discovery of a Tyr-ADPr diagnostic peak (Figure S2C) enhances the confidence of Tyr-ADPr site mapping.

Canonical H3 Histone Marks Reduce the Efficiency of H3S10ADPr on H3 Peptide

We observed that removal of the positively charged Lys through the synthesis of an H3 peptide containing an Ala in position 9 instead of an Lys almost completely abolished Ser-ADPr (Figures 1B and 1C). It is known that acetylation neutralizes the positive charge of Lys residues, whereas methylation maintains the charge. Thus, the presence of this frequently modified residue in our consensus motif led us to hypothesize that modifications of the Lys preceding the Ser may have different effects on Ser-ADPr, a potential mechanism of interplay between the known histone modifications in the H3S10 environment and H3S10ADPr. Additionally, the recent evidence of PARPs conjugating ADPr to phosphorylated DNA (Talhaoui et al., 2016; Munnur and Ahel, 2017) raised the intriguing possibility of PARPs ADPr a phosphorylated peptide—H3S10ph in this case. Because these endogenous histone PTMs (histone marks) are highly dynamic in cells and organisms, an interplay is likely to have important biological consequences. By examining “marked” histone peptides *in vitro*, we can generate “snapshots” of this dynamic interplay.

We therefore set out to investigate the effect on H3S10ADPr of the histone mark environment around H3S10, which is a particularly PTM-rich and biologically important histone region (Huang et al., 2015). H3K9ac severely inhibits histone H3S10ADPr (Figure 3A), as also shown in a recent report (Liszczak et al., 2018), and reversal of Lys9 acetylation by using deacetylase enzymes (HDAC2, SIRT2) re-established this peptide as a substrate for Ser-ADPr by PARP1/HPF1 (Figure S4A). In comparison, H3K9me1 causes only a very mild reduction of H3S10ADPr levels compared to the unmodified peptide. Phosphorylation of the target residue, Ser10, completely blocked ADPr of the peptide, confirming that ADPr and phosphorylation of the Ser10 site are mutually exclusive (Figure 3A). In agreement with this, we did not find any mass spectrometric evidence for ADPr of a phosphorylated Ser. This indicates that PARP1-mediated ADPr of DNA on a phosphate group (Talhaoui et al., 2016; Munnur and Ahel, 2017) is mechanistically different from HPF1-dependent ADPr by PARP1 on protein substrates.

Given that H3K9me1 did not notably compromise S10ADPr levels, we analyzed whether dimethylations or trimethylations, both commonly observed in the histone code, would have a greater impact on the reaction. We noted a stepwise decrease in H3S10ADPr levels on H3K9me1, H3K9me2, and H3K9me3 substrates, with H3K9me3 permitting only a very modest degree of PARP1/HPF1-dependent H3S10ADPr (Figure 3B). Because H3K9me and H3S10ADPr modifications could coexist on the H3 peptide, we investigated whether the recently identified enzyme that removes Ser-ADPr, ARH3, could still access and remove H3S10ADPr in the presence of H3K9me. Our analysis showed that ARH3 was still active against H3S10ADPr, irrespective of the H3K9me marks, and could efficiently erase H3S10ADPr signals from modified H3 peptides (Figure S4B).

To investigate the effect of known histone marks in a wider context, we broadened the scope of our analysis of residues surrounding H3S10ADPr by testing H3K4ac, H3K4me3, H3K14ac, H3K18ac, and H3K18me3 peptides. Of these additional histone marks, only H3K14ac notably affected the subsequent addition of ADPr to H3S10 (Figure 3C).

Figure 2. Discovery of Tyrosine as a Target Residue for ADPr

(A) Autoradiogram showing ADPr of H3 peptide (1–20aa) with Ser10 substituted by Ala, Thr, Tyr, Glu, and Asp, alongside Lys9 substituted by Arg and Ala. Coomassie staining of the SDS-PAGE is included.

(B) High-resolution ETD fragmentation spectrum of an HPF1 peptide modified by ADP-ribose on tyrosine 238. The chemical structure of ADP-ribose is depicted (see also Figure S2B). *1, peaks corresponding to co-isolated species in their original charge state. Multiple species in charge states 2–5 passed through the quadrupole and could not be completely deconvoluted.

(C) High-resolution ETD fragmentation spectrum of an HPF1 peptide modified by ADP-ribose on serine 97. The chemical structure of ADP-ribose is depicted.

(D) Autoradiogram showing a panel of PARPs incubated with HPF1 protein. Reaction with mono(ADP-ribosyl)ating PARP1 E988Q (EQ) mutant enhances detection of the HPF1 ADPr. Coomassie staining of the SDS-PAGE is included.

(E) Autoradiogram showing PARP1 E988Q-mediated ADPr of HPF1 WT, HPF1 R239A, and GST-HPF1 proteins. Coomassie staining of the SDS-PAGE is included.

(F) 293T cells were transfected with the same amount of EV or plasmid expressing WT, S97A, or Y238A FLAG-tagged HPF1 protein and left untreated or treated for 10 or 120 min with H_2O_2. Inputs and FLAG-IPs were analyzed by western blotting. CMV, cytomegalovirus.

(G) 293T parental or PARP1 KO cells were transfected with the same amount of EV or plasmid expressing WT FLAG-tagged HPF1 protein and left untreated or treated for 10 or 120 min with H_2O_2. Inputs and FLAG-IPs were analyzed by western blotting.

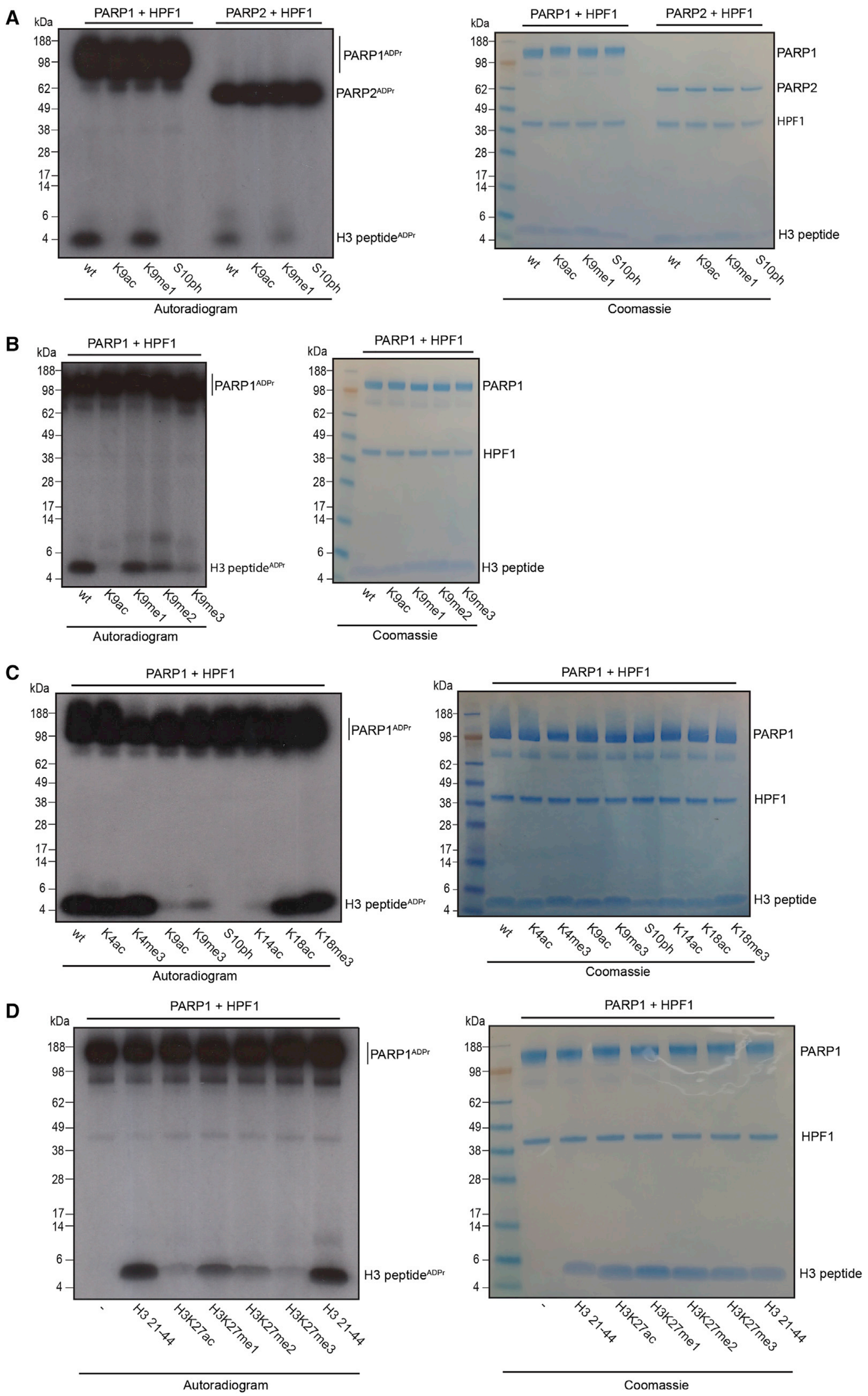

(legend on next page)

Because our earlier experiments had determined that H4 1–23 and H3 27–45 peptides were suitable for PARP1/HPF1-dependent Ser-ADPr modification, we tested both for crosstalk between nearby acetylation and methylation modifications with H4S1ADPr and H3S28ADPr. We found that the modification of the KS motif at S28 has effects similar to those seen for Ser10 (Figure 3D). Alternatively, H3K36me1, H3K36me2, or H3K36me3 did not reduce the H3S28ADPr modification signals, while the H3K36ac had only a modest effect (Figure S4C). We also found that none of the H4R3me2, H4K5ac, or H4K8ac marks had a discernible effect on H4S1ADPr levels compared to the unmodified peptide (Figure S4C).

Ser-ADPr on H3S10 Prevents the Efficient Incorporation of H3K9 Acetylation and H3S10 Phosphorylation

We conducted reciprocal experiments based on our above findings, this time modifying histone H3 peptide first with PARP1/HPF1 complex (Figure S5), then subsequently incubating the reaction products in acetylation, phosphorylation, and methylation reaction mixtures using the purified catalytic domain of p300, the activated fragment of Aurora B kinase (Baronase; to phosphorylate H3S10) and Dim5 methyltransferase (Nunes Bastos et al., 2013; Moonat et al., 2013; Zhang et al., 2003). We detected the acetylated products of Ser-ADPr H3 peptides using a specific H3K9ac antibody and observed that K9ac is effectively prevented if the peptide is previously ADPr (Figure 4A, lane 5). To control for any possible interference of ADPr with western blot detection, we incubated the Ser-ADPr H3 peptide with p300, stopped the reaction, and removed Ser-ADPr from the peptide using ARH3. This assay showed only a negligible amount of H3K9Ac (Figure 4A, lane 6). In a similar experiment, we saw that Ser-ADPr of H3 peptide prevented subsequent H3S10 phosphorylation (Figure 4B, lane 5). Finally, we incubated an Ser-ADPr H3 peptide in an Lys methylation reaction and found that H3S10ADPr did not preclude the incorporation of H3K9me3, although it did substantially reduce the efficiency of the reaction compared to the unmodified H3 peptide (Figure 4C, lane 3 versus lane 5).

An Approach for Rapid and Easy Analysis of ADPr Peptides

Our approaches above use [^{32}P]NAD as a detection method, but this radioactive technique is expensive and requires strict safety procedures. Furthermore, using [^{32}P]NAD and standard gel electrophoresis only allows the detection of modified product, rather than an analysis of unmodified and modified peptides together (i.e., substrates and products). These limitations, together with the clear importance of studying the interplay of Ser-ADPr and other known histone marks, motivated us to look for a simpler approach that could be implemented in virtually any biological laboratory. Given that ADP-ribose is a nucleotide, we reasoned that an electrophoresis system capable of resolving a one-nucleotide difference in the length of oligonucleotides would allow a clear separation of ADPr and unmodified substrate peptides. However, the negatively charged nucleic acids are separated by migrating toward the positively charged anode. In contrast, the histone tail peptides have a net positive charge, even when modified by ADP-ribose and would therefore migrate in the wrong direction. By changing the polarity of the electrodes, the positively charged substrate peptides can be driven into gels intended for electrophoresis of short nucleic acids and be separated according to their charge. Following ADPr, peptides become less positively charged and therefore migrate more slowly, which allows a clear spatial separation between the bands of the modified and unmodified peptides (Figure 5A). After the run, both species (unmodified and modified) can be clearly visualized and quantified by Coomassie-based staining, which reveals by band shift how much of the starting peptide has been ADPr (Figure 5A). Figure 5B shows an exemplar of this technique, comparing unmodified H3 peptide during a time course with PARP1, HPF1, and H3 peptide, with the modified peptide shifted upward at later time points. Incubating modified H3 peptide with ARH3 reverses the band shift to the unmodified state (Figure 5C). We then expanded this method to investigate ADPr efficiency on H3 peptides with a variety of histone marks. We observed that H3K4me mildly reduced ADPr levels compared to WT, whereas H3R8me peptides were modified efficiently (Figure 5D). H3K9ac, H3K9me, and K14ac modification profiles were comparable to the [^{32}P]NAD experiments, reinforcing the value of this Coomassie-based approach for estimating the efficiency of a reaction. Additionally, we examined an H3T11ph peptide, which showed only a very slight ADPr band, suggesting a strong inhibition of HPF1/PARP1-catalyzed Ser-ADPr by the adjacent phosphorylation (Figure 5D). These combined experiments produced a map of the histone marks within a local region around H3S10 that affect the efficiency of H3S10ADPr (Figure 5E). Notably, histone marks other than ADPr also generated a band shift compared to the unmodified counterpart peptide (Figure 5D, left). This implies that the utility of our approach is not limited to ADPr and that this technique can be used to study the dynamics of other histone marks at the peptide level, such as the interplay between phosphorylation and acetylation (Latham and Dent, 2007).

H3K9ac and S10ADPr Are Mutually Exclusive Histone Marks in Human Cells

We sought to assess whether the results generated using histone H3 peptides could be replicated with intact human nucleosomes *in vitro*. WT and H3K9ac recombinant human mononucleosomes were incubated with PARP1 in the presence and

Figure 3. Canonical H3 Histone Marks Reduce the Efficiency of H3S10ADPr on H3 Peptide

(A) Autoradiogram showing PARP1/2 + HPF1-mediated ADPr of H3 peptide with WT, K9ac, K9me1, and S10ph modifications. Coomassie staining of the SDS-PAGE is included.

(B) As in (A), except PARP1 and HPF1 only, with H3 (1–20aa) WT, K9ac, K9me1, K9me2, and K9me3 peptides. Coomassie staining of the SDS-PAGE is included.

(C) As in (B), except with H3 (1–20aa) WT, K4ac, K4me3, K9ac, K9me1, K9me3, S10ph, K14ac, K18ac, and K18me3 peptides. Coomassie staining of the SDS-PAGE is included.

(D) As in (B), except with H3 (21–44aa) WT, K27ac, K27me1, K27me2, K27me3, and WT peptides. Coomassie staining of the SDS-PAGE is included.

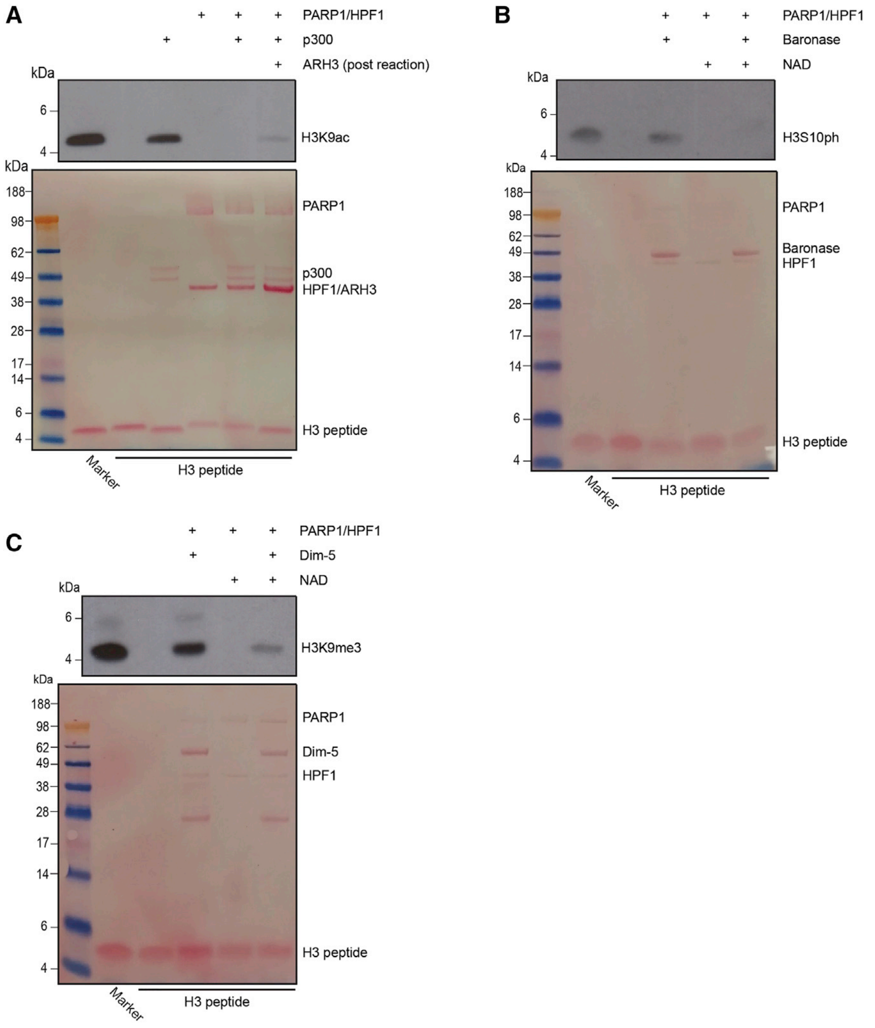

Figure 4. H3S10ADPr Reduces the Efficiency of Subsequent H3K9 Acetylation and H3S10 Phosphorylation

(A) Western blot showing PARP1/HPF1 ADPr of H3 (1–20aa) peptide and subsequent p300-mediated acetylation. One reaction was stopped after p300 incubation, then supplemented with ARH3 to remove ADPr before signal detection. Membrane probed with H3K9ac antibody, with H3K9ac peptide included as a positive marker.

(B) Western blot showing PARP1/HPF1 ADPr of H3 (1–20aa) peptide and subsequent Baronase-mediated phosphorylation. Control sample excludes NAD from the PARP1/HPF1 reaction. Membrane probed with H3S10ph antibody, with H3S10ph peptide included as a positive marker.

(C) Western blot showing PARP1/HPF1 ADPr of H3 (1–20aa) peptide and subsequent Dim5-mediated methylation. Control sample excludes NAD from the PARP1/HPF1 reaction. Membrane probed with H3K9me3 antibody, with H3K9me3 peptide included as a positive marker.

absence of HPF1. We observed a clear contrast between the WT and H3K9ac nucleosomes when incubated with PARP1/HPF1, with WT displaying a higher level of Ser-ADPr (Figure 6A). The H3K9ac nucleosomes were still significantly modified, albeit to a lower degree, presumably due to modifications of other previously observed histone tail sites, such as H3S28 and H2BS6 (Leidecker et al., 2016). Similarly, we performed an assay using a nucleosome substrate to test the reciprocal reactions, namely Ser-ADPr, and then acetylation of the nucleosome (Figure 6B). We saw that prior Ser-ADPr reduced subsequent H3K9 acetylation, as detected by the specific anti-H3K9ac antibody (Figure 6B). These results suggest that the interplay that we observe between Ser-ADPr and acetylation of neighboring Lys residues on the peptide level also occurs in the context of whole nucleosomes and *in vivo*. We also observed that prior H3S10 phosphorylation of the nucleosome also significantly reduced subsequent p300-mediated acetylation of H3K9 (Figure 6B).

To compare the interplay observed in our *in vitro* system with that in cells, we analyzed U2OS cell extracts by high-resolution electron-transfer dissociation (ETD) mass spectrometry (Leidecker et al., 2016). We identified H3S10ADPr in the presence of mono-, di-, and trimethylation of H3K9 and with H3K14ac, but never with H3K9ac (Figures 6C and S6A–S6C). Any detection of H3K9ac was in the absence of H3S10ADPr, although we were able to detect H3K9ac in co-existence with marks other than H3S10ADPr, such as H3K14ac (Figure 6D). To test whether our failure to detect H3K9ac and H3S10ADPr together was due to technical limitations, we purified small amounts of H3K9acS10ADPr peptide generated from a highly inefficient reaction and analyzed it by mass spectrometry. We found that we were able to detect both histone marks on the same peptide (Figure S6D), further indicating that the apparent non-coexistence of these marks is due to the mutual exclusivity *in vivo* rather than our technical inability of detecting doubly modified H3K9ac/H3S10ADPr peptides. Our findings define two groups of histone H3 PTMs that can either coexist with or are mutually exclusive to Ser-ADPr (Figure 6E).

To further characterize the interplay between histone Ser-ADPr and other PTMs *in vivo*, we assessed the levels of H3S10ph, H3K9ac, H3K9me3, and several other PTMs around the H3S10ADPr site in 293T cells following DNA damage (Figure 7A). Our results confirmed previously published data showing reduction of H3K9ac in response to DNA damage (Tjeertes et al., 2009), because we also observed striking specific deacetylation of the H3K9 site after 120 min of treatment (Figure 7A). We also observed significant deacetylation of H3K14 under the same conditions (Figure 7A). DNA damage-induced deacetylation of both H3K9 and H3K14 was completely blocked by pre-treatment with a PARP inhibitor, olaparib. We did not observe DNA damage-induced deacetylation of H3K27ac or K36ac, or demethylation of H3K9me3 or H3K27me3, among

OPEN
ACCESS
CellPress

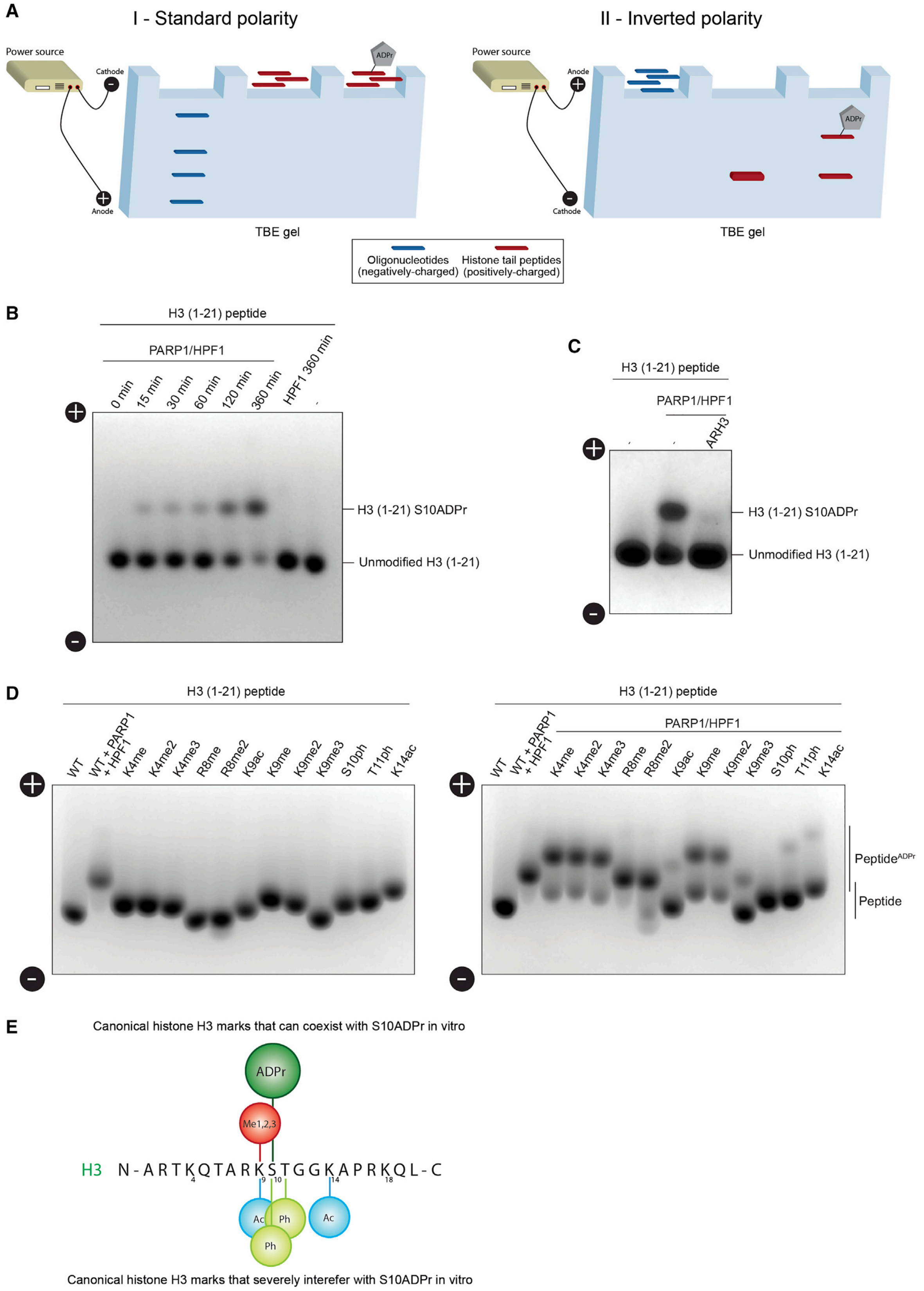

(legend on next page)

others (Figure 7A). As evident from the patterns for the cell-cycle proteins cyclin A, B1, E1, Cdc2 T15P, PRC1 T481P, and p21 (Figure 7A), the cell cycle was unaffected by olaparib treatment in our experimental settings.

To test our hypothesis that Ser-ADPr specifically affects these canonical histone marks, we performed similar experiments in ARH3 KO cells. We have previously demonstrated that these cells have chronically increased histone ADPr, including the Ser10 site (Fontana et al., 2017; Palazzo et al., 2018). DNA damage-induced deacetylation was more robust in these cells, which was especially obvious at 10 and 120 min post-DNA damage (Figure 7B). It is worth mentioning that some other acetylated proteins, detected by pan-acetylation antibody, displayed a different profile of increasing acetylation after DNA damage treatment (Figure 7B). These results combined suggest that interplay between histone ADPr and K9 acetylation and some other forms of histone modifications takes place in living cells. This knowledge can offer a framework for the further investigation of crosstalk between Ser-ADPr and other histone marks, and onward toward a wider understanding of the physiological function of Ser-ADPr as a histone mark and as a PTM.

DISCUSSION

Ser-ADPr is a recent addition to the array of PTMs found on mammalian proteins (Leidecker et al., 2016), and several studies have started to delineate its physiological relevance (Fontana et al., 2017; Bonfiglio et al., 2017b; Palazzo et al., 2018). Ser-ADPr is found on a large number of proteins (Leidecker et al., 2016; Abplanalp et al., 2017; Bonfiglio et al., 2017b), and Ser is the primary amino acid acceptor residue of ADPr following DNA damage in human cells (Palazzo et al., 2018). Histone proteins are subject to a large variety of PTMs, many of which are directly involved in the regulation of chromatin structure and transcription and play important roles in DNA replication and repair (Lawrence et al., 2016). This raises the question as to how Ser-ADPr functions in this densely modified environment and how the other histone marks affect the DDR as a consequence of their crosstalk with Ser-ADPr.

Our findings demonstrate the interplay between Ser-ADPr and a number of canonical histone marks, also showing that the process of Ser-ADPr is dependent on the context of the local histone code and vice versa. Whereas most of the histone Ser-ADPr sites examined in this work have previously been reported to be phosphorylated under certain conditions (Huang et al., 2015; Leidecker et al., 2016), ADPr of H3S10 and H3S28 are particularly intriguing, because H3S10ph and H3S28ph were suggested to play key roles in mitosis (Sawicka and Seiser, 2012; Lawrence et al., 2016). Moreover, acetylation of H3K9 and H3K27, which are mutually exclusive of ADPr at H3S10 and H3S28, respectively, is critical in the regulation of gene expression (Lawrence et al., 2016). This suggests that the prior modification of H3K9 and H3K27 could prevent the expansion of the Ser-ADPr signal or that, following DNA damage, neither H3K9ac/H3K27ac nor H3S10ph/H3S28ph marks would be "permitted" to initiate their responses. Because H3K9ac is frequently associated with transcriptionally active areas (Lawrence et al., 2016), it is logical that in the event of DNA damage, it would be undesirable to collect a large body of repair factors while the transcription machinery is still local and active. Accordingly, in response to DNA damage, H3K9Ac becomes diminished at promoter regions of cell-cycle-responsive and active gene sites and at the sites of DNA lesions (Tjeertes et al., 2009; Bártová et al., 2011; Meyer et al., 2016). Here, we also showed that H3K9ac decreases following H_2O_2 treatment in human cells. The levels of H3K14ac changed in a manner similar to H3K9ac, and K14 acetylation on H3 peptide also blocks H3S10ADPr *in vitro*. Given that H3K9ac and H3K14ac marks have already been shown to have similar patterns and roles in the recruitment of the switch/sucrose non-fermentable (SWI/SNF) chromatin remodeling complex and spreading of γH2AX (Lee et al., 2010), it is not surprising that transient K14ac deacetylation could also be important for efficient DDR.

Notably, we confirmed previous findings that H3S10ph decreases after DNA damage treatment (Monaco et al., 2005), even more sharply than does H3K9ac, suggesting the importance of H3S10 dephosphorylation in DDR. Both DNA damage-induced H3K9 deacetylation and H3S10 dephosphorylation were prevented by treatment with the clinically approved PARP inhibitor olaparib, suggesting that both marks have a strict interplay with PARP1/2 activity. The inhibitory effect of olaparib on H3S10 dephosphorylation was already observed (Monaco et al., 2005). Because H3S10ph is correlated with transcriptional activation, mitosis, and meiosis (Lawrence et al., 2016), it is plausible that DDR-related histone marks could be a detrimental addition during some steps of the cell cycle or in some DNA repair pathways.

Alternatively, the methylation of H3K9 and H3K27, both of which are associated with transcriptional repression (Lawrence et al., 2016; Zhu and Wani, 2010), do not prevent the ADPr of the neighboring Ser sites. In concordance, we showed that cellular H3K9me3 levels remain stable after DNA damage, which was also previously reported (Tjeertes et al., 2009).

Although we did not see the effects of H3K36me2 in our systems, this PTM was previously identified as a DNA damage mark that facilitates the recruitment of DNA repair proteins and

Figure 5. A Technique to Rapidly Analyze ADP-Ribosylated Peptides

(A) Schematic representation of the approach to rapidly and easily analyze the modification status of positively charged histone tail peptides.

(B) Imperial stained gel showing ADPr of H3 (1–21aa) peptides after addition of PARP1/HPF1 during a 6-hr time course. The upward band shift denotes ADPr of the H3 peptide.

(C) Imperial stained gel showing ADPr of H3 (1–21aa) peptides after addition of PARP1/HPF1 and subsequent addition of ARH3. The upward band shift denotes ADPr of the H3 peptide.

(D) Imperial stained gel showing H3 (1–21aa) WT, K4me1, K4me2, K4me3, R8me1, R8me2a, K9ac, K9me1, K9me2, K9me3, S10ph, T11ph, and K14ac peptides and subsequent ADPr following addition of PARP1 and HPF1. The upward band shift denotes ADPr of the H3 peptide.

(E) A schematic showing a map of histone H3 1–20aa with histone marks that interfere with Ser-ADPr on H3 peptide *in vitro*.

OPEN
ACCESS
CellPress

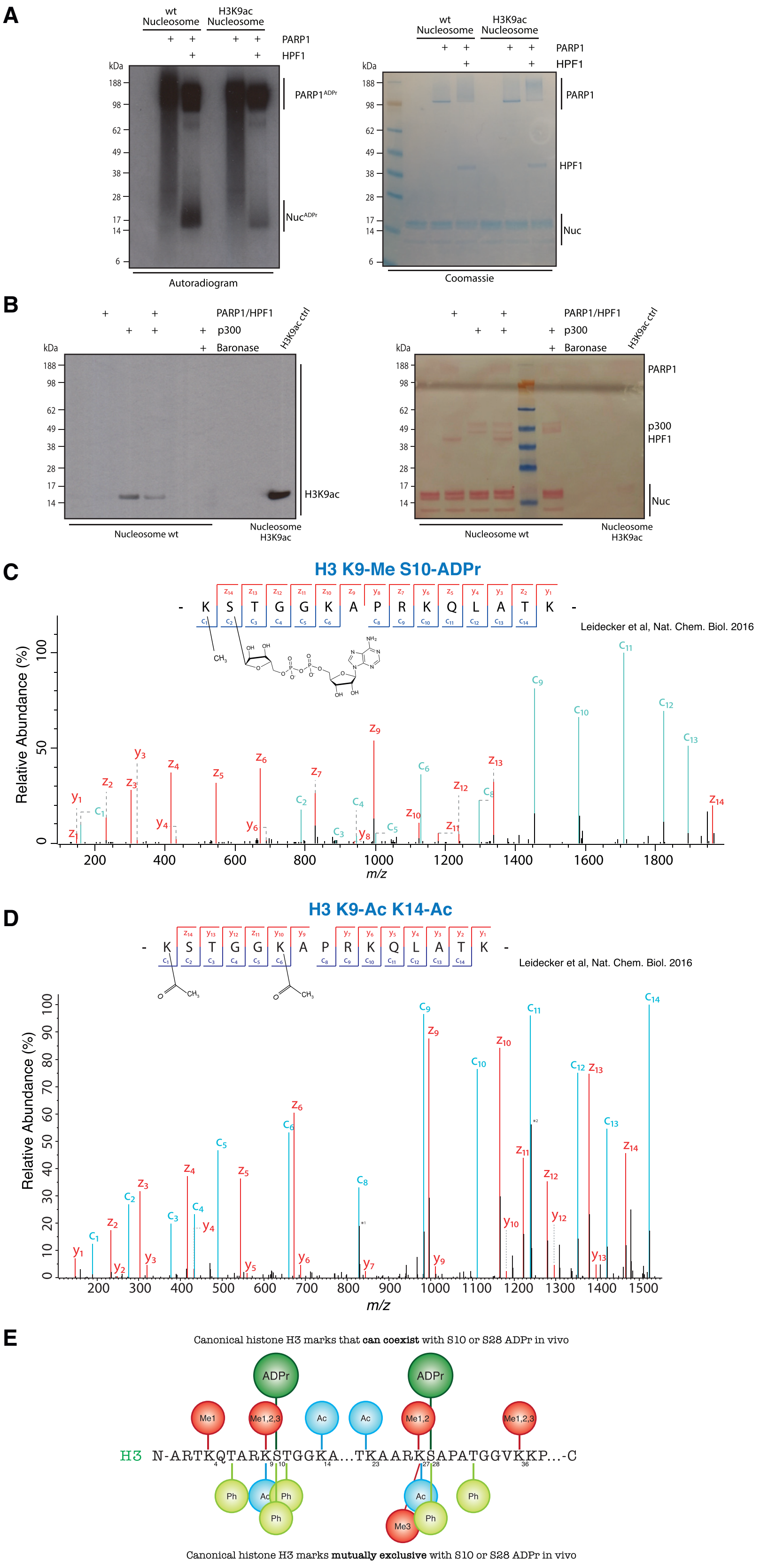

Figure 6. H3K9ac and S10ADPr Are Mutually Exclusive Histone Marks in Human Cells

(A) Autoradiogram showing PARP1 mediated ADPr in the presence of absence of HPF1, with either WT or K9ac human recombinant nucleosomes. Coomassie staining of the SDS-PAGE is included.

(B) Western blot showing PARP1/HPF1 ADPr of recombinant human nucleosome and subsequent p300-mediated acetylation. One reaction includes Baronase incubation instead of ADPr reaction, before p300 acetylation reaction. Membrane probed with H3K9ac antibody, with commercially obtained recombinant human H3K9ac nucleosome included as a positive marker.

(C) High-resolution ETD fragmentation spectrum of a H3 peptide modified by methyl on lysine 9 and ADP-ribose on serine 10 obtained from Leidecker et al. (2016). The chemical structure of methyl and ADP-ribose are depicted.

(D) High-resolution ETD fragmentation spectrum of a H3 peptide modified by acetylation on lysine 9 and lysine 14 obtained from Leidecker et al. (2016). The chemical structure of acetylation is depicted. *1, Peak corresponding to an unfragmented co-eluting, co-isolated +2 precursor deconvoluted into the +1 state. *2, Peak corresponding to an unfragmented co-eluting, co-isolated +3 precursor deconvoluted into the +1 state.

(E) Schematic summary of canonical histone H3 marks and their interactions with Ser-ADPr based on the mass spectrometry (MS) analysis of U2OS cell extracts from Leidecker et al. (2016). The marks depicted on the top are H3 marks that can coexist with Ser10 or Ser28 ADPr *in vivo*, while the H3 marks depicted on the bottom are mutually exclusive with ADPr on Ser10 or Ser28.

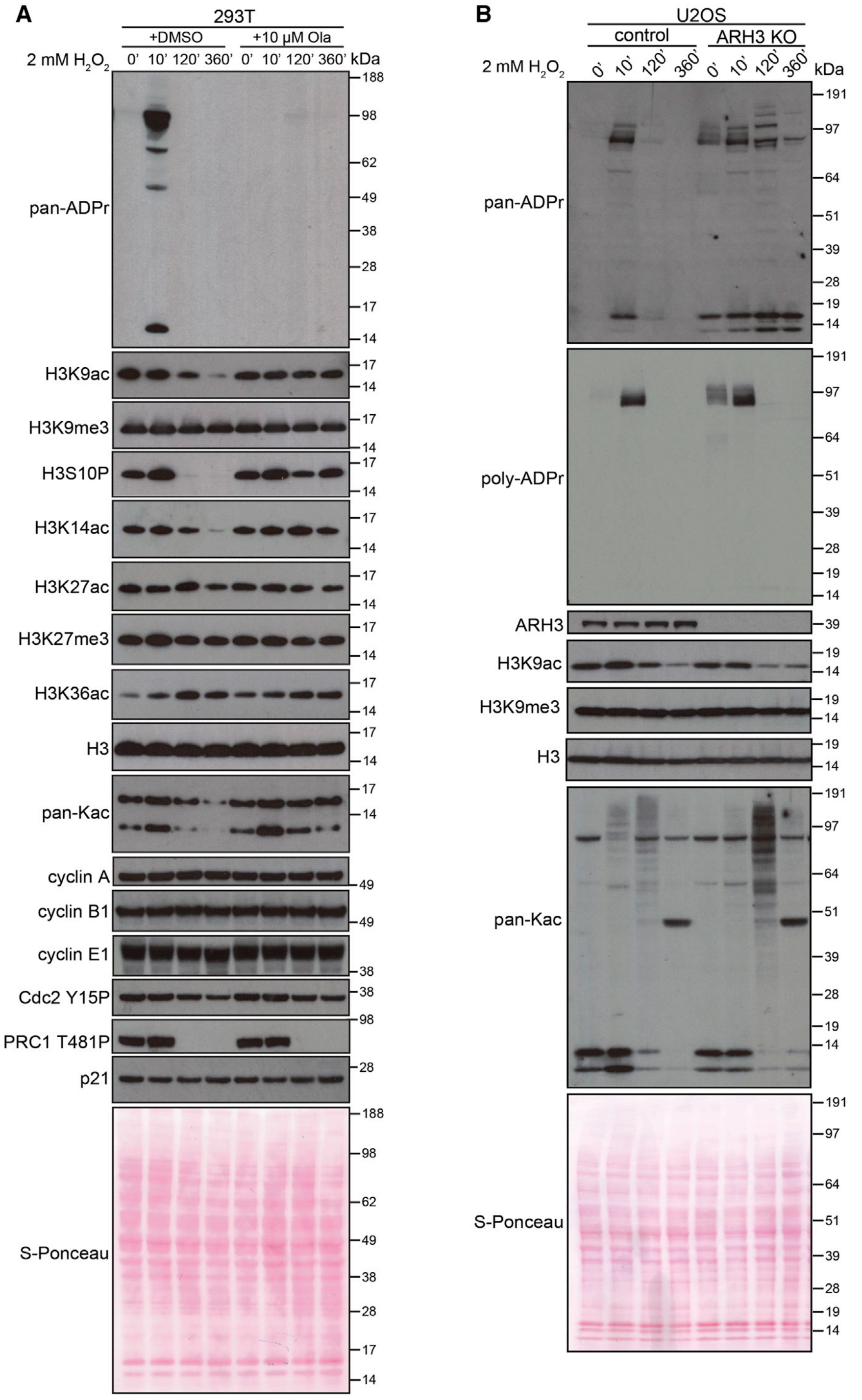

Figure 7. Histone Mark Response to DNA Damage with PARP Inhibition and Persistent Ser-ADPr

(A) 293T cells were pretreated with DMSO or olaparib and treated with H_2O_2. Western blotting analysis of the changes in histone H3 K9ac, K9me3, S10P, K14ac, K27ac, K27me3, and K36ac, as well as total pan-Kac histone acetylation and cell-cycle protein levels was performed at the indicated times after the induction of DNA damage.

(B) U2OS WT and ARH3 KO cells were treated with H_2O_2. The levels of H3K9ac, H3K9me3, and pan-Kac were examined by western blotting at the indicated time points.

is crucial for efficient repair (Fnu et al., 2011). Changes in many other histone modifications have also been described during the DDR. Of note, PARP1 can change PTM landscapes, not only by means of ADPr but also indirectly by modulating the activity of chromatin remodelers and histone-modifying enzymes (Gupte et al., 2017). According to our results, not all of the histone marks are involved in the interplay with Ser-ADPr following a certain DNA damage stimulus. However, these histone marks may be involved in response to other agents and/or in control of different DNA repair pathways. Nevertheless, it is tempting to speculate that it is the specific combination of many histone PTMs that defines the exact molecular pathway that the cell will follow to attempt DNA repair.

The inhibitory effect of histone acetylation on Ser-ADPr could explain why combining PARP inhibitors (PARPis) with histone deacetylase inhibitors (HDACis) results in increased DNA damage and cellular sensitivity (Min et al., 2015; Chao and Goodman, 2014; Rasmussen et al., 2016; Konstantinopoulos et al., 2014; Zhang et al., 2012; Kim et al., 2017). While both PARPis and HDACis are already individually in clinical trials, our data showing that DNA damage-induced K9 deacetylation is blocked by PARPis further strengthen the pre-clinical rationale for their simultaneous administration. At the same time, future investigation of the effects of including HDACis into the known combination therapies involving PARPis (Dréan et al., 2016) could highlight other promising therapeutic strategies.

The field of Ser-ADPr has developed rapidly due to advances in proteomics methods to overcome longstanding technical challenges associated with unbiased ADPr mapping (Leidecker et al., 2016; Bonfiglio et al., 2017a, 2017b). Beyond Ser-ADPr site identification, future efforts will be directed toward understanding the functional consequences of Ser-ADPr and the detailed molecular mechanisms. ADPr differs among the well-studied PTMs for its lack of experimental tools and techniques needed for progress in the field. This is beginning to change (Crawford et al., 2018), and to facilitate the investigation of Ser-ADPr, we have also developed approaches that can be readily adopted by the majority of

biological laboratories (Fontana et al., 2017; Palazzo et al., 2018). Here, we have also introduced a method to dramatically simplify and improve the detection of Ser-ADPr peptides. This strategy overcomes the limitations of traditional radioactive techniques and allows an estimation of the extent of modification, which is impossible with other available techniques. Thus, our approach will clearly facilitate investigations of the dynamics of canonical histone marks, such as phosphorylation and acetylation.

We have demonstrated that Ser-ADPr is a histone mark that is mutually exclusive with neighboring acetylation and phosphorylation both *in vitro* and *in vivo*. Further characterizing the interplay between Ser-ADPr and the PTMs of histone residues (Liszczak et al., 2018) and of other proteins is of great interest and will provide valuable insights into the complex crosstalk regulating the architecture and accessibility of chromatin. Given the conservation of the KS motifs in Ser-ADPr for hundreds of proteins, acetylation and phosphorylation of the KS motifs could likely be a general strategy to regulate ADPr of proteins involved in genome stability beyond histones.

This study and the tools we have developed have also led to the discovery of Tyr-ADPr (also noted recently by Leslie Pedrioli et al., 2018). As with the recent discovery of Ser-ADPr, this initial finding poses a tantalizing number of questions. While we show that PARP1/HPF1 is able to catalyze Tyr-ADPr, are there other writers of this modification? What are enzymes that remove Tyr-ADPr? How common is this modification and what is the physiological relevance? The HPF1 Tyr-ADPr site (Tyr238) constitutes the main PARP1-binding residue (Gibbs-Seymour et al., 2016), and substitution of Tyr238 (along with Arg239) to Ala prevents PARP1/HPF1 interaction. These data suggest that Tyr238 modification could be a secondary level of regulation for the stability and activity of the PARP1/HPF1 complex. In this context, our discovery of a Tyr-ADPr diagnostic peak will enhance future proteomics identifications of Tyr-ADPr sites and understanding of their physiological relevance. In summary, our study provides insights into the interplay of Ser-ADPr and other histone marks and provides evidence for an intriguing type of ADPr.

STAR★METHODS

Detailed methods are provided in the online version of this paper and include the following:

- KEY RESOURCES TABLE
- CONTACT FOR REAGENT AND RESOURCE SHARING
- EXPERIMENTAL MODEL AND SUBJECT DETAILS
- METHODS DETAILS
  - *In vitro* ADPr assays
  - Reanalysis of published high-quality proteomics datasets
  - Western blotting
  - Immunoprecipitation experiments
- QUANTIFICATION AND STATISTICAL ANALYSIS

SUPPLEMENTAL INFORMATION

Supplemental Information includes six figures and can be found with this article online at https://doi.org/10.1016/j.celrep.2018.08.092.

ACKNOWLEDGMENTS

Many thanks to Pietro Fontana, Francis Barr, and Rob Klose for the kind gift of proteins used in this study, and to Ian Gibbs-Seymour, Thomas Agnew, and Luca Palazzo for their advice and comments on the manuscript. The work in the Ahel laboratory was funded by the Wellcome Trust (grants 101794 and 210634), Cancer Research UK (grant C35050/A22284), and the European Research Council (grant 281739). E.B. is funded by the Kyoto Institute of Technology and the Japan Society for the Promotion of Science. The work in the Matic laboratory was funded by the Deutsche Forschungsgemeinschaft (Cellular Stress Responses in Aging-Associated Diseases) (grant EXC 229 to I.M.) and the European Union's Horizon 2020 research and innovation program (Marie Skłodowska-Curie grant agreement 657501 to J.J.B. and I.M.).

AUTHOR CONTRIBUTIONS

I.M., J.J.B., E.B., and I.A. conceived the study. E.B. designed and performed the biochemical studies. J.J.B. and T.C. acquired and analyzed the mass spectrometric data. J.J.B., T.C., and I.M. designed and J.J.B. performed the inverted polarity native gel analysis. E.P. and F.Z. performed the cell biology experiments. E.B. wrote the manuscript, with contributions from all of the authors.

DECLARATION OF INTERESTS

The authors declare no competing interests.

Received: April 2, 2018
Revised: June 29, 2018
Accepted: August 30, 2018
Published: September 25, 2018

REFERENCES

Abplanalp, J., Leutert, M., Frugier, E., Nowak, K., Feurer, R., Kato, J., Kistemaker, H.V.A., Filippov, D.V., Moss, J., Caflisch, A., and Hottiger, M.O. (2017). Proteomic analyses identify ARH3 as a serine mono-ADP-ribosylhydrolase. Nat. Commun. *8*, 2055.

Barkauskaite, E., Jankevicius, G., and Ahel, I. (2015). Structures and mechanisms of enzymes employed in the synthesis and degradation of PARP-dependent protein ADP-ribosylation. Mol. Cell *58*, 935–946.

Bártová, E., Šustáčková, G., Stixová, L., Kozubek, S., Legartová, S., and Foltánková, V. (2011). Recruitment of Oct4 protein to UV-damaged chromatin in embryonic stem cells. PLoS One *6*, e27281.

Bilan, V., Leutert, M., Nanni, P., Panse, C., and Hottiger, M.O. (2017). Combining higher-energy collision dissociation and electron-transfer/higher-energy collision dissociation fragmentation in a product-dependent manner confidently assigns proteomewide ADP-ribose acceptor sites. Anal. Chem. *89*, 1523–1530.

Bonfiglio, J.J., Colby, T., and Matic, I. (2017a). Mass spectrometry for serine ADP-ribosylation? Think o-glycosylation!. Nucleic Acids Res. *45*, 6259–6264.

Bonfiglio, J.J., Fontana, P., Zhang, Q., Colby, T., Gibbs-Seymour, I., Atanassov, I., Bartlett, E., Zaja, R., Ahel, I., and Matic, I. (2017b). Serine ADP-ribosylation depends on HPF1. Mol. Cell *65*, 932–940.e6.

Chao, O.S., and Goodman, O.B., Jr. (2014). Synergistic loss of prostate cancer cell viability by coinhibition of HDAC and PARP. Mol. Cancer Res. *12*, 1755–1766.

Cohen, M.S., and Chang, P. (2018). Insights into the biogenesis, function, and regulation of ADP-ribosylation. Nat. Chem. Biol. *14*, 236–243.

Cox, J., and Mann, M. (2008). MaxQuant enables high peptide identification rates, individualized p.p.b.-range mass accuracies and proteome-wide protein quantification. Nat. Biotechnol. *26*, 1367–1372.

Cox, J., Neuhauser, N., Michalski, A., Scheltema, R.A., Olsen, J.V., and Mann, M. (2011). Andromeda: a peptide search engine integrated into the MaxQuant environment. J. Proteome Res. *10*, 1794–1805.

Crawford, K., Bonfiglio, J.J., Mikoč, A., Matic, I., and Ahel, I. (2018). Specificity of reversible ADP-ribosylation and regulation of cellular processes. Crit. Rev. Biochem. Mol. Biol. *53*, 64–82.

Dréan, A., Lord, C.J., and Ashworth, A. (2016). PARP inhibitor combination therapy. Crit. Rev. Oncol. Hematol. *108*, 73–85.

Dunstan, M.S., Barkauskaite, E., Lafite, P., Knezevic, C.E., Brassington, A., Ahel, M., Hergenrother, P.J., Leys, D., and Ahel, I. (2012). Structure and mechanism of a canonical poly(ADP-ribose) glycohydrolase. Nat. Commun. *3*, 878.

Fnu, S., Williamson, E.A., De Haro, L.P., Brenneman, M., Wray, J., Shaheen, M., Radhakrishnan, K., Lee, S.H., Nickoloff, J.A., and Hromas, R. (2011). Methylation of histone H3 lysine 36 enhances DNA repair by nonhomologous end-joining. Proc. Natl. Acad. Sci. USA *108*, 540–545.

Fontana, P., Bonfiglio, J.J., Palazzo, L., Bartlett, E., Matic, I., and Ahel, I. (2017). Serine ADP-ribosylation reversal by the hydrolase ARH3. eLife *6*, e28533.

Gibbs-Seymour, I., Fontana, P., Rack, J.G.M., and Ahel, I. (2016). HPF1/C4orf27 is a PARP-1-interacting protein that regulates PARP-1 ADP-ribosylation activity. Mol. Cell *62*, 432–442.

Gupte, R., Liu, Z., and Kraus, W.L. (2017). PARPs and ADP-ribosylation: recent advances linking molecular functions to biological outcomes. Genes Dev. *31*, 101–126.

Huang, H., Lin, S., Garcia, B.A., and Zhao, Y. (2015). Quantitative proteomic analysis of histone modifications. Chem. Rev. *115*, 2376–2418.

Kim, Y., Kim, A., Sharip, A., Sharip, A., Jiang, J., Yang, Q., and Xie, Y. (2017). Reverse the resistance to PARP inhibitors. Int. J. Biol. Sci. *13*, 198–208.

Konstantinopoulos, P.A., Wilson, A.J., Saskowski, J., Wass, E., and Khabele, D. (2014). Suberoylanilide hydroxamic acid (SAHA) enhances olaparib activity by targeting homologous recombination DNA repair in ovarian cancer. Gynecol. Oncol. *133*, 599–606.

Langelier, M.F., Planck, J.L., Servent, K.M., and Pascal, J.M. (2011). Purification of human PARP-1 and PARP-1 domains from Escherichia coli for structural and biochemical analysis. Methods Mol. Biol. *780*, 209–226.

Langelier, M.F., Riccio, A.A., and Pascal, J.M. (2014). PARP-2 and PARP-3 are selectively activated by 5′ phosphorylated DNA breaks through an allosteric regulatory mechanism shared with PARP-1. Nucleic Acids Res. *42*, 7762–7775.

Latham, J.A., and Dent, S.Y.R. (2007). Cross-regulation of histone modifications. Nat. Struct. Mol. Biol. *14*, 1017–1024.

Lawrence, M., Daujat, S., and Schneider, R. (2016). Lateral thinking: how histone modifications regulate gene expression. Trends Genet. *32*, 42–56.

Lee, H.S., Park, J.H., Kim, S.J., Kwon, S.J., and Kwon, J. (2010). A cooperative activation loop among SWI/SNF, gamma-H2AX and H3 acetylation for DNA double-strand break repair. EMBO J. *8*, 1434–1445.

Leidecker, O., Bonfiglio, J.J., Colby, T., Zhang, Q., Atanassov, I., Zaja, R., Palazzo, L., Stockum, A., Ahel, I., and Matic, I. (2016). Serine is a new target residue for endogenous ADP-ribosylation on histones. Nat. Chem. Biol. *12*, 998–1000.

Leslie Pedrioli, D.M., Leutert, M., Bilan, V., Nowak, K., Gunasekera, K., Ferrari, E., Imhof, R., Malmström, L., and Hottiger, M.O. (2018). Comprehensive ADP-ribosylome analysis identifies tyrosine as an ADP-ribose acceptor site. EMBO Rep. *19*, 8.

Liszczak, G., Diehl, K.L., Dann, G.P., and Muir, T.W. (2018). Acetylation blocks DNA damage-induced chromatin ADP-ribosylation. Nat. Chem. Biol. *14*, 837–840.

Martello, R., Leutert, M., Jungmichel, S., Bilan, V., Larsen, S.C., Young, C., Hottiger, M.O., and Nielsen, M.L. (2016). Proteome-wide identification of the endogenous ADP-ribosylome of mammalian cells and tissue. Nat. Commun. *7*, 12917.

Martin-Hernandez, K., Rodriguez-Vargas, J.-M., Schreiber, V., and Dantzer, F. (2017). Expanding functions of ADP-ribosylation in the maintenance of genome integrity. Semin. Cell Dev. Biol. *63*, 92–101.

Matic, I., Ahel, I., and Hay, R.T. (2012). Reanalysis of phosphoproteomics data uncovers ADP-ribosylation sites. Nat. Methods *9*, 771–772.

Mehrotra, P.V., Ahel, D., Ryan, D.P., Weston, R., Wiechens, N., Kraehenbuehl, R., Owen-Hughes, T., and Ahel, I. (2011). DNA repair factor APLF is a histone chaperone. Mol. Cell *41*, 46–55.

Meyer, B., Fabbrizi, M.R., Raj, S., Zobel, C.L., Hallahan, D.E., and Sharma, G.G. (2016). Histone H3 lysine 9 acetylation obstructs ATM activation and promotes ionizing radiation sensitivity in normal stem cells. Stem Cell Reports *7*, 1013–1022.

Min, A., Im, S.A., Kim, D.K., Song, S.H., Kim, H.J., Lee, K.H., Kim, T.Y., Han, S.W., Oh, D.Y., Kim, T.Y., et al. (2015). Histone deacetylase inhibitor, suberoylanilide hydroxamic acid (SAHA), enhances anti-tumor effects of the poly (ADP-ribose) polymerase (PARP) inhibitor olaparib in triple-negative breast cancer cells. Breast Cancer Res. *17*, 33.

Monaco, L., Kolthur-Seetharam, U., Loury, R., Murcia, J.M., de Murcia, G., and Sassone-Corsi, P. (2005). Inhibition of Aurora-B kinase activity by poly(ADP-ribosyl)ation in response to DNA damage. Proc. Natl. Acad. Sci. USA *102*, 14244–14248.

Moonat, S., Sakharkar, A.J., Zhang, H., Tang, L., and Pandey, S.C. (2013). Aberrant histone deacetylase2-mediated histone modifications and synaptic plasticity in the amygdala predisposes to anxiety and alcoholism. Biol. Psychiatry *73*, 763–773.

Munnur, D., and Ahel, I. (2017). Reversible mono-ADP-ribosylation of DNA breaks. FEBS J. *284*, 4002–4016.

Nunes Bastos, R., Gandhi, S.R., Baron, R.D., Gruneberg, U., Nigg, E.A., and Barr, F.A. (2013). Aurora B suppresses microtubule dynamics and limits central spindle size by locally activating KIF4A. J. Cell Biol. *202*, 605–621.

Palazzo, L., Daniels, C.M., Nettleship, J.E., Rahman, N., McPherson, R.L., Ong, S., Kato, K., Nureki, O., Leung, A.K.L., and Ahel, I. (2016). ENPP1 processes protein ADP-ribosylation in vitro. FEBS J. *283*, 3371–3388.

Palazzo, L., Mikoč, A., and Ahel, I. (2017). ADP-ribosylation: new facets of an ancient modification. FEBS J. *284*, 2932–2946.

Palazzo, L., Leidecker, O., Prokhorova, E., Dauben, H., Matic, I., and Ahel, I. (2018). Serine is the major residue for ADP-ribosylation upon DNA damage. eLife *7*, e34334.

Pascal, J.M., and Ellenberger, T. (2015). The rise and fall of poly(ADP-ribose): an enzymatic perspective. DNA Repair (Amst.) *32*, 10–16.

Posavec Marjanović, M., Crawford, K., and Ahel, I. (2017). PARP, transcription and chromatin modeling. Semin. Cell Dev. Biol. *63*, 102–113.

Rack, J.G.M., VanLinden, M.R., Lutter, T., Aasland, R., and Ziegler, M. (2014). Constitutive nuclear localization of an alternatively spliced sirtuin-2 isoform. J. Mol. Biol. *426*, 1677–1691.

Rasmussen, R.D., Gajjar, M.K., Jensen, K.E., and Hamerlik, P. (2016). Enhanced efficacy of combined HDAC and PARP targeting in glioblastoma. Mol. Oncol. *10*, 751–763.

Sawicka, A., and Seiser, C. (2012). Histone H3 phosphorylation - a versatile chromatin modification for different occasions. Biochimie *94*, 2193–2201.

Talhaoui, I., Lebedeva, N.A., Zarkovic, G., Saint-Pierre, C., Kutuzov, M.M., Sukhanova, M.V., Matkarimov, B.T., Gasparutto, D., Saparbaev, M.K., Lavrik, O.I., and Ishchenko, A.A. (2016). Poly(ADP-ribose) polymerases covalently modify strand break termini in DNA fragments in vitro. Nucleic Acids Res. *44*, 9279–9295.

Tan, M., Luo, H., Lee, S., Jin, F., Yang, J.S., Montellier, E., Buchou, T., Cheng, Z., Rousseaux, S., Rajagopal, N., et al. (2011). Identification of 67 histone marks and histone lysine crotonylation as a new type of histone modification. Cell *146*, 1016–1028.

Tjeertes, J.V., Miller, K.M., and Jackson, S.P. (2009). Screen for DNA-damage-responsive histone modifications identifies H3K9Ac and H3K56Ac in human cells. EMBO J. *28*, 1878–1889.

Vivelo, C.A., and Leung, A.K. (2015). Proteomics approaches to identify mono-(ADP-ribosyl)ated and poly(ADP-ribosyl)ated proteins. Proteomics *15*, 203–217.

Vyas, S., Matic, I., Uchima, L., Rood, J., Zaja, R., Hay, R.T., Ahel, I., and Chang, P. (2014). Family-wide analysis of poly(ADP-ribose) polymerase activity. Nat. Commun. *5*, 4426.

Zhang, X., Tamaru, H., Khan, S.I., Horton, J.R., Keefe, L.J., Selker, E.U., and Cheng, X. (2002). Structure of the Neurospora SET domain protein DIM-5, a histone H3 lysine methyltransferase. Cell *111*, 117–127.

Zhang, X., Yang, Z., Khan, S.I., Horton, J.R., Tamaru, H., Selker, E.U., and Cheng, X. (2003). Structural basis for the product specificity of histone lysine methyltransferases. Mol. Cell *12*, 177–185.

Zhang, L., Qin, X., Zhao, Y., Fast, L., Zhuang, S., Liu, P., Cheng, G., and Zhao, T.C. (2012). Inhibition of histone deacetylases preserves myocardial performance and prevents cardiac remodeling through stimulation of endogenous angiomyogenesis. J. Pharmacol. Exp. Ther. *341*, 285–293.

Zhu, Q., and Wani, A.A. (2010). Histone modifications: crucial elements for damage response and chromatin restoration. J. Cell. Physiol. *223*, 283–288.

STAR★METHODS

KEY RESOURCES TABLE

REAGENT or RESOURCE	SOURCE	IDENTIFIER
Chemicals, Peptides, and Recombinant Proteins		
Recombinant human mononucleosomes	EpiCypher	Cat# 16-0006
Recombinant human mononucleosomes H3 K9ac	EpiCypher	Cat# 16-0314
Activated DNA	Trevigen	Cat# 4671-096-06
NAD+	Trevigen	Cat# 4684-096-02
32P-NAD+	Hartmann Analytic	Cat# ARP 0141
Histone H3 (1 - 21), Biotinylated	AnaSpec	Cat# AS-61702
Histone H3K4me (1 - 21), Biotinylated	AnaSpec	Cat# AS-64355
Histone H3K4me2 (1 - 21), Biotinylated	AnaSpec	Cat# AS-64356
Histone H3K4me3 (1 - 21), Biotinylated	AnaSpec	Cat# AS-64357
Histone H3R8me (1 - 21), Biotinylated	AnaSpec	Cat# AS-64607
Histone H3R8me2 (1 - 21), Biotinylated	AnaSpec	Cat# AS-64972
Histone H3R8me2 (1 - 21), Biotinylated	AnaSpec	Cat# AS-64972
Histone H3K9ac (1 - 21), Biotinylated	AnaSpec	Cat# AS-64361
Histone H3K9me (1 - 21), Biotinylated	AnaSpec	Cat# AS-64358
Histone H3K9me2 (1 - 21), Biotinylated	AnaSpec	Cat# AS-64359
Histone H3K9me3 (1 - 21), Biotinylated	AnaSpec	Cat# AS-64360
Histone H3S10ph (1 - 21), Biotinylated	AnaSpec	Cat# AS-64611
Histone H3T11ph (1 - 21), Biotinylated	AnaSpec	Cat# AS-64973
Histone H3K14ac (1 - 21), Biotinylated	AnaSpec	Cat# AS-64362
Histone H3 peptide 1-20aa, biotinylated	EpiCypher	Cat# 12-0001
Recombinant human PARP1 protein	Langelier et al., 2011	N/A
Recombinant human PARP2 protein	Langelier et al., 2014	N/A
Histone H3 peptide K4ac 1-20aa, biotinylated	EpiCypher	Cat# 12-0002
Histone H3 peptide K14ac 1-20aa, biotinylated	EpiCypher	Cat# 12-0004
Histone H3 peptide K18ac 1-20aa, biotinylated	EpiCypher	Cat# 12-0005
Histone H3 peptide K4me3 1-20aa, biotinylated	EpiCypher	Cat# 12-0009
Histone H3 peptide K18me3 1-20aa, biotinylated	EpiCypher	Cat# 12-0015
Histone H3 peptide K9ac 1-20aa, biotinylated	EpiCypher	Cat# 12-0003
Histone H3 peptide K9me1 1-20aa, biotinylated	EpiCypher	Cat# 12-0010
Histone H3 peptide K9me2 1-20aa, biotinylated	EpiCypher	Cat# 12-0011
Histone H3 peptide K9me3 1-20aa, biotinylated	EpiCypher	Cat# 12-0012
Histone H3 peptide S10ph 1-20aa, biotinylated	EpiCypher	Cat# 12-0041
Histone H3 peptide 15-34aa, biotinylated	EpiCypher	Cat# 12-0016
Histone H3 peptide 27-45aa, biotinylated	EpiCypher	Cat# 12-0020
Histone H4 peptide 1-23aa, biotinylated	EpiCypher	Cat# 12-0029
Histone H4 peptide K5ac 1-23aa, biotinylated	EpiCypher	Cat# 12-0030
Histone H4 peptide K8ac 1-23aa, biotinylated	EpiCypher	Cat# 12-0031
Histone H4 peptide R3me 1-23aa, biotinylated	EpiCypher	Cat# 12-0059
Histone H2A peptide 1-17aa, biotinylated	EpiCypher	Cat# 12-0112
Histone H3 peptide K36ac 27-45aa, biotinylated	EpiCypher	Cat# 12-0129
Histone H3 peptide K36me1 27-45aa, biotinylated	EpiCypher	Cat# 12-0174
Histone H3 peptide K36me2 27-45aa, biotinylated	EpiCypher	Cat# 12-0115
Histone H3 peptide K36me3 27-45aa, biotinylated	EpiCypher	Cat# 12-0212

(*Continued on next page*)

Continued

REAGENT or RESOURCE	SOURCE	IDENTIFIER
Recombinant human HPF1 protein	Gibbs-Seymour et al., 2016	N/A
Recombinant human HPF1 Y238F protein	This paper	N/A
Recombinant human HPF1 Y238A protein	This paper	N/A
Recombinant human HPF1 Y238E protein	This paper	N/A
Recombinant human ARH3 protein	Fontana et al., 2017	N/A
Imperial Protein Stain	Thermofisher Scientific	Cat# 24615
Olaparib	Cayman Chemical	Cat# 10621
Histone H3 (21-44), Biotinylated	AnaSpec	Cat# AS-64641
Histone H3K27ac (21-44), Biotinylated	AnaSpec	Cat# AS-64637
Histone H3 (21-44), Biotinylated	AnaSpec	Cat# AS-64440
Histone H3K27me1 (21-44), Biotinylated	AnaSpec	Cat# AS-64365
Histone H3K27me2 (21-44), Biotinylated	AnaSpec	Cat# AS-64366
Histone H3K27me3 (21-44), Biotinylated	AnaSpec	Cat# AS-64367
Histone H3/H4 tetramer	Mehrotra et al., 2011	N/A
p300, human, recombinant, catalytic domain	Enzo Life Sciences	Cat# BML-SE451-0100
Dim-5, recombinant, Neurospora crassa	Zhang et al., 2002	N/A
Baronase	Gift from Francis Barr (University of Oxford)	N/A
PARP3, human, recombinant	Bonfiglio et al., 2017b	N/A
PARP10 catalytic domain, human, recombinant	Palazzo et al., 2016	N/A
Recombinant human PARP1 E988Q protein	Langelier et al., 2011	N/A
HDAC2, human recombinant	Active Motif	Cat# 31505
SIRT2, human, recombinant	Rack et al., 2014	N/A
Antibodies		
anti-pan-ADP-ribose (rabbit monoclonal)	Millipore	Cat# MABE1016; RRID:AB_2665466
anti-PAR (rabbit polyclonal)	Trevigen	Cat# 4336-BPC-100; RRID:AB_2721257
anti-Flag HRP-conjugated (mouse monoclonal)	Sigma-Aldrich	Cat# A8592; RRID:AB_439702
anti-HPF1 (rabbit polyclonal)	Gibbs-Seymour et al., 2016	N/A
anti-Flag M2 agarose-conjugated (mouse monoclonal)	Sigma-Aldrich	Cat#: A2220; RRID:AB_1006303
anti-ARH3/ADPRH (rabbit polyclonal)	Atlas Antibodies	Cat#: HPA027104; RRID:AB_1060133
anti-histone H3, CT, pan (rabbit polyclonal)	Millipore	Cat#: 07-690; RRID:AB_417398
anti-H3K9ac (rabbit monoclonal)	Cell Signaling	Cat#: 9649; RRID:AB_823528
anti-H3K14ac (rabbit monoclonal)	Cell Signaling	Cat#: 7627S; RRID:AB_1083941
anti-H3K27ac (rabbit monoclonal)	Cell Signaling	Cat#: 8173P; RRID:AB_1094988
anti-H3K36ac (rabbit monoclonal)	Cell Signaling	Cat#: 27683
anti-H3K9me3 (rabbit polyclonal)	Abcam	Cat#: ab8898; RRID:AB_306848
anti-H3K27me3 (rabbit polyclonal)	Gift from Rob Klose (University of Oxford)	N/A
anti-pan-Kac (rabbit polyclonal)	Cell Signaling	Cat#: 9441; RRID:AB_331805
anti-H3S10P (rabbit polyclonal)	Abcam	Cat#: ab5176; RRID:AB_304763
anti-cyclin A (rabbit polyclonal)	Santa Cruz Biotechnology	Cat#: sc-751; RRID:AB_631329
anti-cyclin B1 (rabbit polyclonal)	Millipore	Cat#: 05-373; RRID:AB_309701
anti-cyclin E1 (mouse monoclonal)	Cell Signaling	Cat#: 4129; RRID:AB_2071200
anti-PRC1 T481P (rabbit monoclonal)	Abcam	Cat#: ab62366; RRID:AB_944969
anti-Cdc2 Y15P (rabbit monoclonal)	Cell Signaling	Cat#: 4539S; RRID:AB_560953
anti-p21 (rabbit polyclonal)	Santa Cruz Biotechnology	Cat#: sc-397; RRID:AB_632126
anti-pan-Kac (rabbit polyclonal)	Abcam	Cat#: ab21623; RRID:AB_446436
anti-PARP1 (rabbit monoclonal)	Abcam	Cat#: ab32138; RRID:AB_777101

(*Continued on next page*)

Continued

REAGENT or RESOURCE	SOURCE	IDENTIFIER
Experimental Models: Cell Lines		
Human: U2OS cells	ATCC	Cat# HTB-96
Human: U2OS ARH3 KO cells	Fontana et al., 2017	N/A
Human: 293T cells	ATCC	Cat# CRL-3216
Human: 293T HPF1 KO cells	Gibbs-Seymour et al., 2016	N/A
Human: 293T PARP1 KO cells	Gift from John Pascal (University Montreal)	N/A
Recombinant DNA		
pDONR221 (Gateway vector)	Thermo Fisher Scientific	12536017
Flag-H3.1 WT (plasmid)	Palazzo et al., 2018	N/A
Flag-H3.1 S10A (plasmid)	Palazzo et al., 2018	N/A
Flag-H3.1 K9A (plasmid)	This paper	N/A
Flag-H3.1 K9R (plasmid)	This paper	N/A
Flag-H3.1 K9Q (plasmid)	This paper	N/A
Flag-HPF1 WT (plasmid)	Gibbs-Seymour et al., 2016	N/A
Flag-HPF1 Y238A (plasmid)	Gibbs-Seymour et al., 2016	N/A
Flag-HPF1 Y238F (plasmid)	This paper	N/A
Flag-HPF1 S97A (plasmid)	This paper	N/A
Flag C3X-EV (Gateway vector)	Gibbs-Seymour et al., 2016	N/A
Flag CMV-EV (Gateway vector)	Gibbs-Seymour et al., 2016	N/A
Software and Algorithms		
MaxQuant proteomics suite of algorithms (version 1.5.3.17)	Cox and Mann, 2008	http://www.coxdocs.org/doku.php?id=maxquant:start
Deposited Data		
Mass spectrometry data: MS analysis of endogenous histones	Leidecker et al., 2016	ProteomeXchange: PXD005462
Mass spectrometry data: enrichment of modified peptides with a macrodomain ADPr-binding module	Martello et al., 2016	ProteomeXchange: PXD004245
Mass spectrometry data: cellular ADP-ribosylome characterization with HCD and EThcD	Bilan et al., 2017	ProteomeXchange: PXD004676

CONTACT FOR REAGENT AND RESOURCE SHARING

Further information and requests for resources and reagents should be directed to and will be fulfilled by the Lead Contact, Ivan Ahel (ivan.ahel@path.ox.ac.uk).

EXPERIMENTAL MODEL AND SUBJECT DETAILS

Most of the experiments in these studies utilized recombinant protein and enzymes, as well as chemically synthesized peptides. For the cell biology experiments we used standard human model cell lines U2OS (ATCC HTB-96; osteosarcoma) and HEK293T (ATCC CRL-3216; embryonic kidney). The cells were grown in DMEM (Sigma-Aldrich) supplemented with 10% FBS (GIBCO) and penicillin-streptomycin (100 U/ml, GIBCO) at 37°C with 5% CO_2. The generation of ARH3 KO U2OS cells was previously described (Fontana et al., 2017). Absence of mycoplasma contamination confirmed by MycoAlert Mycoplasma Detection Kit.

METHODS DETAILS

In vitro ADPr assays

A variety of *in vitro* ADPr assays were used to measure the ability of enzymes to modify or demodify different substrates.

Recombinant proteins and peptides

Recombinant proteins are purified as described previously (Langelier et al., 2011; Langelier et al., 2014; Gibbs-Seymour et al., 2016; Fontana et al., 2017; Dunstan et al., 2012). Peptides were purchased from EpiCypher or custom made. Nucleosomes were from EpiCypher.

Enzymatic preparation of the modified histone peptides

Recombinant Dim-5 (the homolog of human SUV39H1/2) and SIRT2 were purified as previously described (Rack et al., 2014; Zhang et al., 2002). Recombinant p300 was purchased from Enzo Life Sciences. HDAC2 was purchased from Active Motif. For histone phosphorylation reactions we used the activated Aurora B fragment called Baronase, which was a gift from the Barr lab (Nunes Bastos et al., 2013). H3 peptides were purchased from EpiCypher. H3 peptides (either WT or Ser-ADPr modified as described above) were incubated in either; HAT buffer (p300) - 50 mM Tris-HCl pH 8.0, 1mM DTT, 100 μM Acetyl-CoA, 10% glycerol for 30 min at 30°C; phosphorylation buffer (Baronase) - 50 mM Tris-HCl pH 7.5, 10 mM $MgCl_2$, 1 mM ATP, 10 mM DTT for 60 min at 37°C; methyltransferase buffer (Dim-5,) – 50mM Glycine pH 9.8, 2 mM DTT, 10% glycerol for 20 min at room temperature. Reactions were then analyzed by SDS-PAGE and western blotting (detailed below). HDACi reactions (HDAC2, SIRT2) were performed in reaction buffer contained 50 mM Tris-HCl pH 8.0, 100 mM NaCl, 2 mM $MgCl_2$, which were subsequently supplemented by PARP1/HPF1, activated DNA and 50 μM NAD^+ spiked with $^{32}PNAD^+$. The modification reaction proceeded at room temperature for 20 min before addition of the PARPi Olaparib at 1 μM. Reactions were then analyzed by SDS-PAGE and autoradiography.

Standard radioactivity-based ADPr assay

Recombinant proteins or peptides were ADPr by PARP1 in the presence or absence of HPF1 and histone peptides. PARP1 concentration in the assays was 1 μM unless stated otherwise, HPF1 was always equimolar to PARP1, histone peptides were used at 0.5 μg per reaction, and recombinant nucleosomes were at 1 μM. The PARP reaction buffer contained 50 mM Tris-HCl pH 8.0, 100 mM NaCl, 2 mM $MgCl_2$, activated DNA and 50 μM NAD^+ spiked with $^{32}PNAD^+$. The modification reaction proceeded at room temperature for 20 min before addition of the PARPi Olaparib at 1 μM. Reactions were then analyzed by SDS-PAGE and autoradiography.

Inverted polarity native gels for ADPr detection

This simple, non-radioactive method allows visualization of both substrates and products of ADPr reactions. ADPr reactions were performed as described above, except with 2 μg histone peptide per reaction, and in the presence of non-radioactive NAD^+. Samples were mixed with TBE Sample Buffer and loaded on 20% TBE gels in TBE Running Buffer. Samples were run with inverted polarity at 200 V for 1 hr. Gels were then fixed for 30 min in 10% glutaraldehyde, washed in H_20 for 3 × 10 min, then stained with Imperial Protein Stain for 1 hr.

In vitro *ADP-ribosyl glycohydrolase assays*

The assays were performed as in Fontana et al., 2017. Briefly, H3 peptides were incubated with PARP1 and HPF1, under the conditions described above, and stopped by addition of Olaparib. ARH3 was then added to the reactions for incubation at room temperature for 30 min. Reactions were then analyzed by SDS-PAGE and autoradiography. ARH3 concentration was at 1 μM.

Reanalysis of published high-quality proteomics datasets

For the reanalysis of published high-quality proteomics datasets, public raw files were analyzed with MaxQuant proteomics suite of algorithms (version 1.5.3.17) (Cox and Mann, 2008), using the integrated search engine Andromeda (Cox et al., 2011).

Data from the published proteomics study of peptides enriched with an ADPr-binding macrodomain (Martello et al., 2016) were searched against the human proteome database (downloaded 09.10.2015 from UniProt) with the following parameters: the maximum allowed mass deviation was set to 4.5 ppm for precursor ions and 20 ppm for fragment ions; the minimum peptide length was set to 6 amino acids and the maximum number of missed cleavages was set to 5 with the maximum charge state 7. Variable modifications included acetylation (Protein N-term), Oxidation (M) and ADPr (DEKRSTCYNQHM). The variable modification ADPr allowed for neutral losses of adenine (m/z 136.0618); adenosine with loss of water (m/z 250.0935); AMP (m/z 348.0704); ADP (m/z 428.0367) and ADP-ribose (m/z 542.0684). FTMS top peaks per 100 Da were set to 20. We employed the annotated mass spectrometry (MS)/MS spectra generated by MaxQuant as the basis for our manual validation of spectra. To consider a peptide as modified on Tyr, we required the presence of fragment ions with either the intact ADP-ribose or phosphoribose (resulting from the loss of AMP) pointing to ADPr on Tyr. Unmodified "native" sequence ions were not considered as evidence for localization since it is impossible to distinguish between an original lack of modification and complete loss of ADPr during fragmentation. Two additional pieces of evidence supporting Tyr modification could also be observed in lower mass regions of these spectra. First, a peak matching the immonium ion of modified Tyr (+ ADPr – AMPloss) could be observed (albeit weakly) in these spectra at 330.0742 Da (+1). The native (unmodified) Tyr immonium ion (136.0762) was also generally very weak (~5%) in comparison to the immediately neighboring Adenine peak (136.0623) in these spectra, as opposed to those of peptides containing Tyr but with ADPR on serine. The significance of this ratio as support of Tyr modification can only be fully assessed with larger numbers of ETD-verified peptide spectra.

For the cellular ADPr characterization with HCD and EThcD study (Bilan et al., 2017), variable modifications included oxidation (M), acetylation (Protein N-term and K) and ADPr (DEKRSTYCMNQHM). For confident identification of ADPr sites, we considered only ETD MS/MS spectra and required a minimum Andromeda score of 100, mass deviation smaller than 3 ppm after MaxQuant recalibration and a localization score above 0.9. In addition, we manually validated all the representative spectra by requiring extensive coverage of the peptide backbone fragment ions. For localization we required the clear presence of multiple high-intensity fragment ions pinpointing the modification site.

For the cellular MS analysis of endogenous histones study (Leidecker et al., 2016), variable modifications included oxidation (M), acetylation (Protein N-term and K), methylation (KR), dimethylation (K), trimethylation (K) and ADPr (DEKRSTYCMNQHM). Similarly, we considered only ETD MS/MS spectra and required a minimum Andromeda score of 100, mass deviation smaller than 3 ppm after MaxQuant recalibration and a localization score above 0.9.

Western blotting

Human U2OS (ATCC HTB-96) and HEK293T (ATCC CRL-3216) cells were plated and grown overnight in DMEM (Sigma-Aldrich) supplemented with 10% FBS (GIBCO) and penicillin-streptomycin (100 U/ml, GIBCO). To induce DNA damage, cells were incubated with 2 mM H_2O_2 (Sigma-Aldrich) in DPBS with calcium and magnesium (GIBCO) for the indicated times. For PARPi, cells were pretreated with 10 μM Olaparib for 1 hr, and Olaparib was also added to the DPBS solution in case of the induction of DNA damage. Cells were lysed with Triton X-100 lysis buffer (50 mM Tris-HCl pH 8.0, 100 mM NaCl, 1% Triton X-100) at 4°C. Right before use, the buffer was supplemented with 5 mM $MgCl_2$, protease and phosphatase inhibitors (Roche), 1 μM ADP-HPD and 1 μM Olaparib. Benzonase (Sigma) was added to the cell lysates and incubated for 20 min at 4°C. After centrifugation at 14,000 rpm for 15 min, supernatants were collected. Protein concentrations were analyzed by Bradford Protein Assay (Bio-Rad). Proteins were boiled in NuPAGE LDS sample buffer (Invitrogen), resolved on NuPAGE Novex 4%–12% Bis-Tris gels (Invitrogen), and transferred onto nitrocellulose membranes (Bio-Rad) using Trans-Blot Turbo Transfer System (Bio-Rad). Membranes were blocked in PBS buffer with 0.05% Tween 20 and 5% non-fat dried milk for 1 hr at room temperature, and incubated overnight with primary antibodies at 4°C, followed by 1-hour incubation with peroxidase-conjugated secondary antibodies at room temperature. Blots were developed using ECL (Invitrogen) and analyzed by exposing to films.

Immunoprecipitation experiments

Flag-immunoprecipitation followed by western blotting was used to analyze the modification status of the precipitated proteins and their mutant versions. Full-length human histone H3.1 and HPF1 cDNAs were cloned into the pDONR221 vector (Thermo Fisher Scientific). Point mutations were produced in pDONR-H3.1 and pDONR-HPF1 using QuikChange Lightning site-directed mutagenesis kit (Agilent). Mammalian expression constructs expressed H3.1 proteins with the C-terminal 3xFlag tag, and HPF1 proteins with N-terminal Flag tag. Wild-type proteins and their mutant versions were expressed in 293T cells. The cells were plated, cultured overnight, and transfected using Polyfect (QIAGEN) with an empty vector or a plasmid expressing the Flag-tagged protein of interest for 24 hr essentially as described (Palazzo et al., 2018). The cell lysates were obtained the same as for the western blotting. Protein concentrations were analyzed by Bradford Protein Assay (Bio-Rad), normalized, and then, the cell lysates were incubated with anti-Flag M2 agarose-conjugated mouse monoclonal antibody (Sigma-Aldrich) for 1 hr while rotating at 4°C. Beads were washed several times with Triton X-100 lysis buffer and eluted with NuPAGE LDS sample buffer (Invitrogen). The samples were then analyzed by Western Blotting as described above.

QUANTIFICATION AND STATISTICAL ANALYSIS

The qualitative gel-based assays were used to visualize the experimental results. Representative gels from at least three independent biological replicates were shown.

Cell Reports, Volume *24*

Supplemental Information

Interplay of Histone Marks

with Serine ADP-Ribosylation

Edward Bartlett, Juan José Bonfiglio, Evgeniia Prokhorova, Thomas Colby, Florian Zobel, Ivan Ahel, and Ivan Matic

Figure S1

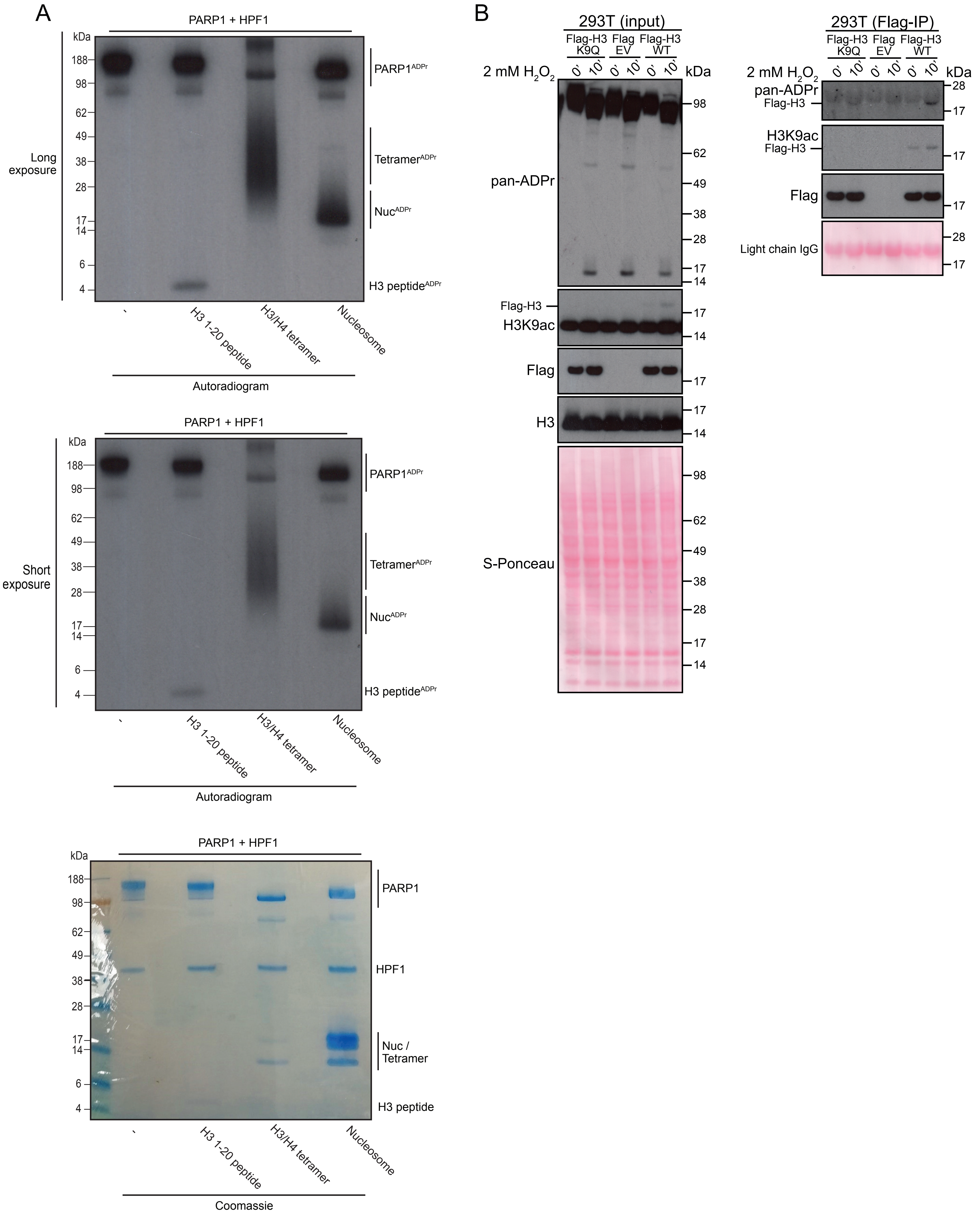

A
PARP1 + HPF1
kDa
188
98
62
49
38
28
17
14
6
4
Long exposure
PARP1^ADPr
Tetramer^ADPr
Nuc^ADPr
H3 peptide^ADPr
-
H3 1-20 peptide
H3/H4 tetramer
Nucleosome
Autoradiogram
PARP1 + HPF1
Short exposure
PARP1^ADPr
Tetramer^ADPr
Nuc^ADPr
H3 peptide^ADPr
-
H3 1-20 peptide
H3/H4 tetramer
Nucleosome
Autoradiogram
PARP1 + HPF1
PARP1
HPF1
Nuc / Tetramer
H3 peptide
-
H3 1-20 peptide
H3/H4 tetramer
Nucleosome
Coomassie
B
293T (input)
Flag-H3 K9Q
Flag EV
Flag-H3 WT
2 mM H2O2
0' 10' 0' 10' 0' 10'
kDa
pan-ADPr
Flag-H3
H3K9ac
Flag
H3
S-Ponceau
293T (Flag-IP)
Flag-H3 K9Q
Flag EV
Flag-H3 WT
2 mM H2O2
pan-ADPr
Flag-H3
H3K9ac
Flag-H3
Flag
Light chain IgG

Figure S1. Related to Figure 1

(A) Autoradiogram showing PARP1/HPF1 mediated ADPr of H3 (1-20aa) peptide, H3/H4 tetramer and recombinant nucleosome. Coomassie staining of the SDS-PAGE is included.

(B) H3K9Q mutant (acetylation mimetic) prevents Ser-ADPr of histone H3 following DNA damage. 293T wt cells were transfected with the same amount of EV or plasmid expressing WT or K9Q Flag-tagged histone H3 protein, and treated for 10 minutes with H_2O_2.

Figure S2

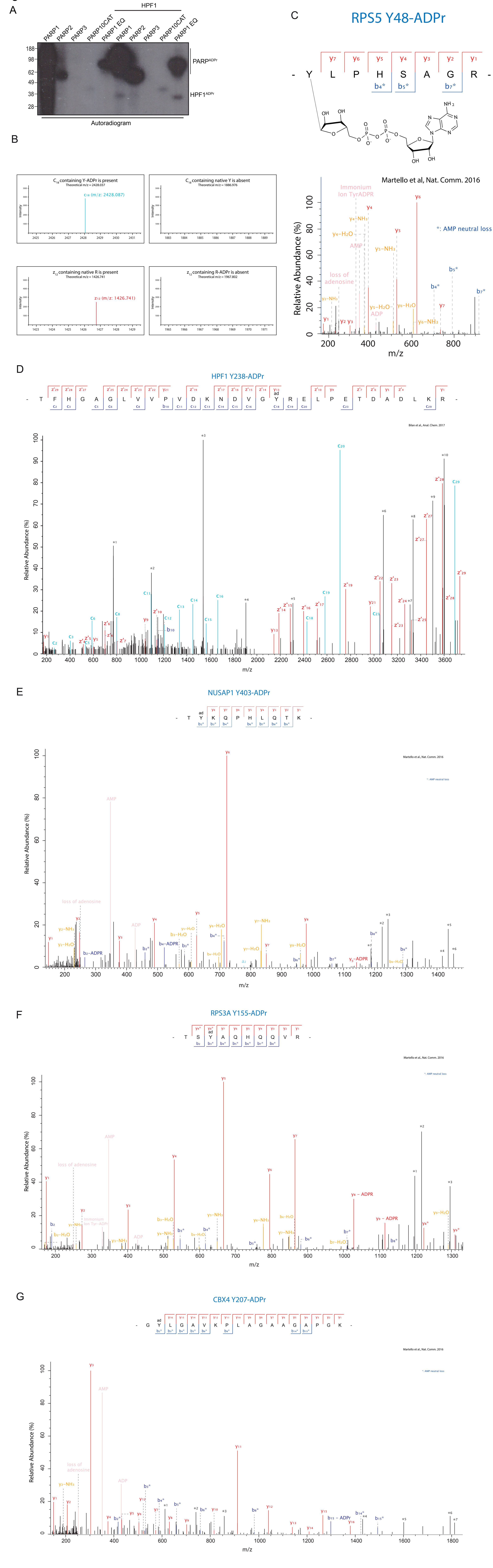
A
HPF1
PARP1
PARP2
PARP3
PARP10CAT
PARP1 EQ
PARP^{ADPr}
HPF1^{ADPr}
Autoradiogram
B
C18 containing Y-ADPr is present
Theoretical m/z = 2428.037
c18 (m/z: 2428.087)
C18 containing native Y is absent
Theoretical m/z = 1886.976
z12 containing native R is present
Theoretical m/z = 1426.741
z12 (m/z: 1426.741)
z12 containing R-ADPr is absent
Theoretical m/z = 1967.802
Intensity
C
RPS5 Y48-ADPr
Martello et al, Nat. Comm. 2016
Immonium Ion TyrADPR
loss of adenosine
*: AMP neutral loss
Relative Abundance (%)
m/z
D
HPF1 Y238-ADPr
Bilan et al., Anal. Chem. 2017
Relative Abundance (%)
m/z
E
NUSAP1 Y403-ADPr
Martello et al., Nat. Comm. 2016
*: AMP neutral loss
loss of adenosine
AMP
ADP
Relative Abundance (%)
m/z
F
RPS3A Y155-ADPr
Martello et al., Nat. Comm. 2016
*: AMP neutral loss
loss of adenosine
AMP
Relative Abundance (%)
m/z
G
CBX4 Y207-ADPr
Martello et al., Nat. Comm. 2016
*: AMP neutral loss
loss of adenosine
AMP
ADP
Relative Abundance (%)
m/z

Figure S2. Discovery of Tyrosine as a target residue for ADPr. Related to Figure 2

(A) A longer exposure of the autoradiogram from Figure 2D, showing a panel of PARPs incubated with HPF1 protein, with visible PARP1 EQ mediated HPF1 ADPr.

(B) Zoom of the spectrum of Figure 2B in different m/z ranges showing the presence of sequence ions that pinpoint ADPr to Y238 (left panels), and the absence of signal in the region that would correspond to sequence ions containing R239-ADPr (right panels).

(C) High-resolution HCD fragmentation spectrum of an RPS5 peptide modified by ADP-ribose obtained from reprocessing a published high-quality proteomics dataset (Martello et al., 2016). Note that the presence of a Tyr-ADPr diagnostic peak in this HCD spectrum (Immonium Ion TyrADPR on the figure) enhances the confidence to assign tyrosine 48 as the modified residue.
* AMP neutral loss. The immonium ion for ADPr-Tyrosine has also undergone the loss of AMP.

(D) High-resolution ETD fragmentation spectrum of a HPF1 peptide modified by ADP-ribose on tyrosine 238 from reprocessing a published high-quality proteomics dataset (Bilan et al., 2017). *[1] peaks corresponding to co-isolated species in their original charge state. Multiple species in charge state 2-5 passed through the quadruopoley and could not be completely deconvoluted. *[2] unassigned peak (m/z = 1098.539). *[3] peak corresponding to an unfragmented co-eluting, co-isolated +2 precursor deconvoluted into the +1 state. *[4] unassigned peak (m/z = 1905.373). *[5] peak corresponding to an unfragmented co-eluting, co-isolated +3 precursor deconvoluted into the +1 state. *[6] peak corresponding to an unfragmented co-eluting, co-isolated +4 precursor deconvoluted into the +1 state. *[7] peak corresponding to the identified +5 precursor after a complete loss of ADPr due to HCD fragmentation*. *[8] peak corresponding to the Z28 ion of the identified +5 precursor after a loss of Adenosine due to HCD fragmentation[a]. *[9] peak corresponding to the identified +5 precursor after a loss of ADP due to HCD fragmentation[a]. *[10] peak corresponding to the identified +5 precursor after a loss of Adenosine due to HCD fragmentation[a].
[a]These features illustrate some disadvantage of supplemental HCD activation. The assigned products illustrate that HCD-induced fragmentation is taking place on the identified modified 5+ peptide and its ETD fragments, thus complicating the spectrum.

(E) High-resolution HCD fragmentation spectra of a NUSAP peptide modified by Tyr-ADP-ribose obtained from reprocessing a published high-quality proteomics dataset (Martello et al., 2016). *[1] peak corresponding to an unfragmented co-eluting, co-isolated +2 precursor deconvoluted into the +1 state. *[2] peak corresponding to the identified precursor after a loss of ADPr and H2O. *[3] peak corresponding to the identified precursor after a loss of ADPr. *[4] peak corresponding to the identified precursor after a loss of AMP and H2O. *[5] peak corresponding to the identified precursor after a loss of AMP. *[6] peak corresponding to the identified precursor after a loss of AMP and an addition of H2O.

(F) High-resolution HCD fragmentation spectra of a RPS3A peptide modified by Tyr-ADP-ribose obtained from reprocessing a published high-quality proteomics dataset (Martello et al., 2016). *[1] peak corresponding to the identified precursor after a loss of ADPr and H2O. *[2] peak corresponding to the identified precursor after a loss of ADPr. *[3] peak corresponding to the identified precursor after a loss of AMP.

(G) High-resolution HCD fragmentation spectra of a CBX4 peptide modified by Tyr-ADP-ribose obtained from reprocessing a published high-quality proteomics dataset (Martello et al., 2016). *[1] peak corresponding to an internal fragment of the precursor (_PLAGAAGA_). *[2] peak corresponding to an internal fragment of the precursor (_KPLAGAAGA_). *[3] peak corresponding to an internal fragment of the precursor (_GAVKPLAGAA_). *[4] peak corresponding to an unfragmented co-eluting, co-isolated +2 precursor deconvoluted into the +1 state. *[5] peak corresponding to the identified precursor after a loss of ADPr. *[6] peak corresponding to the identified precursor after a loss of AMP. *[7] peak corresponding to the identified precursor after a loss of AMP and H2O.

Figure S3

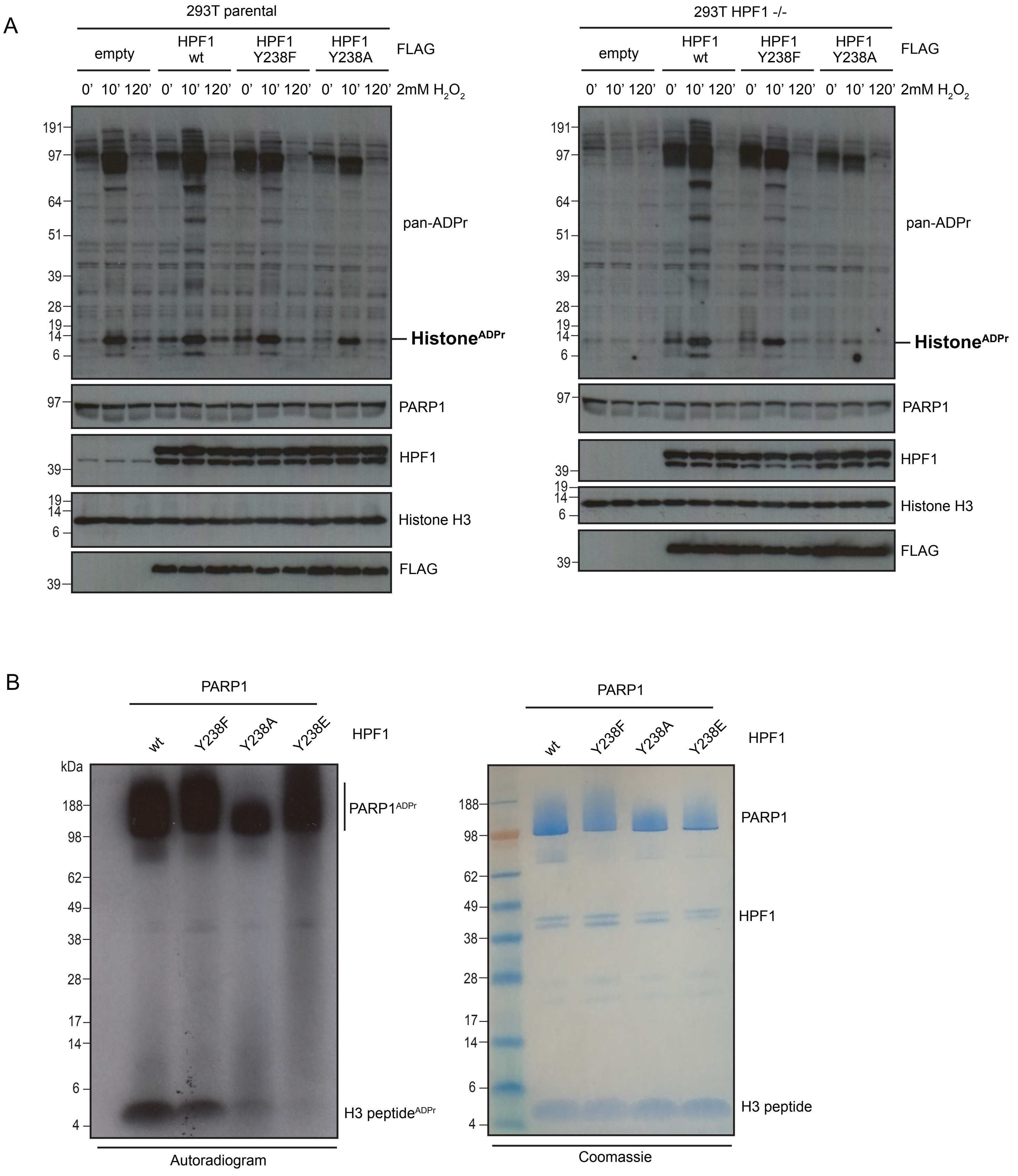

Figure S3. Mutation of HPF1 Tyrosine 238 to phenylalanine supports global Ser-ADP-ribosylation. Related to Figure 2

(A) Complementation of HPF1 in wt and -/- human cells. 293T wt or HPF1 -/- cells were transfected with the same amount of empty vector or plasmid expressing WT, Y238F or Y238A Flag-tagged HPF1 protein, and treated for 0, 10 or 120 minutes with H2O2.

(B) Autoradiogram showing ADPr of H3 peptide following incubation with PARP1 and either HPF1 wt, Y238F, Y238A or Y238E proteins. Coomassie staining of the SDS-PAGE is included.

Figure S4

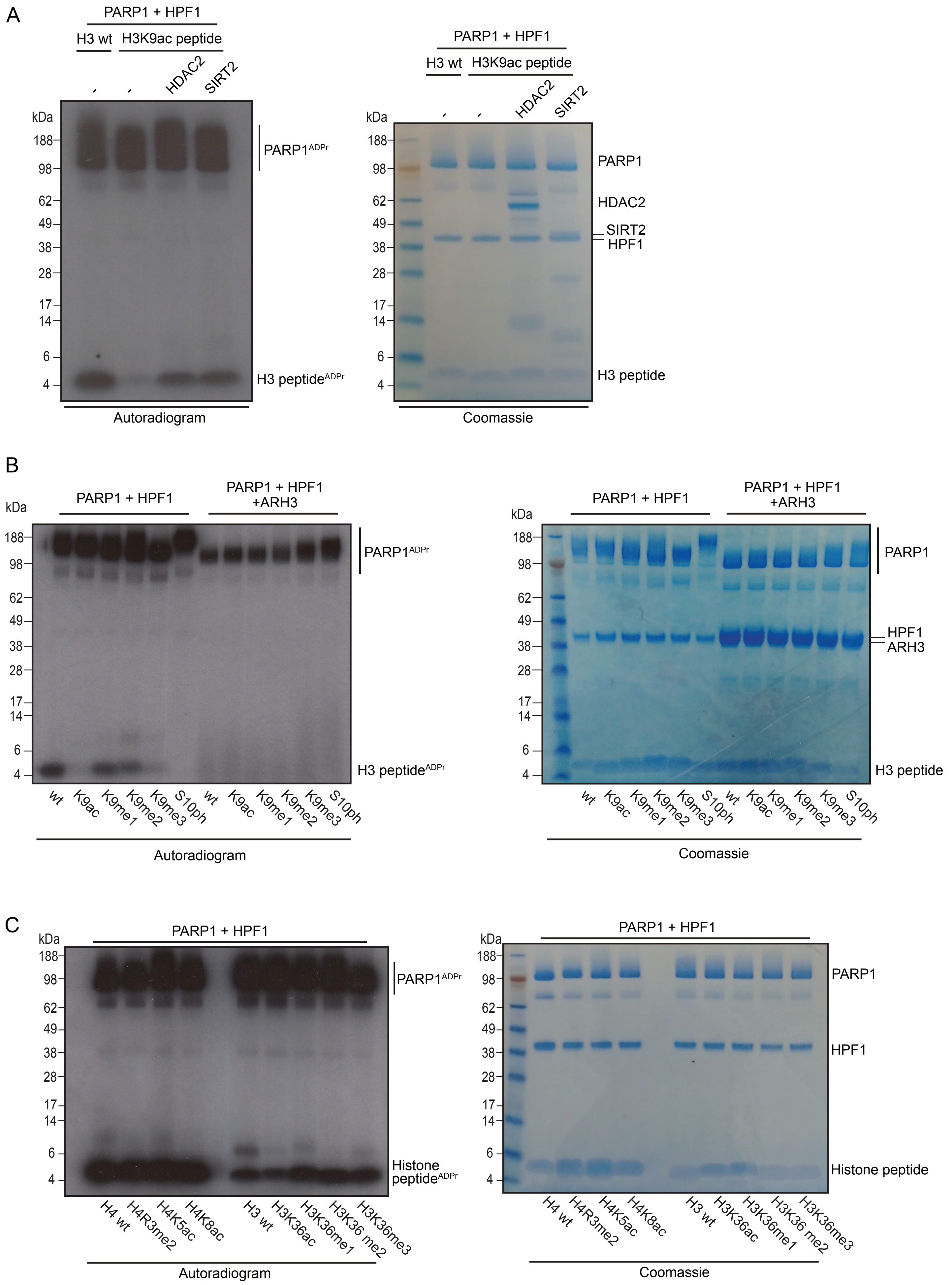
A
PARP1 + HPF1
H3 wt
H3K9ac peptide
HDAC2
SIRT2
kDa
188
98
62
49
38
28
17
14
6
4
PARP1ADPr
H3 peptideADPr
Autoradiogram
PARP1
HDAC2
SIRT2
HPF1
H3 peptide
Coomassie
B
PARP1 + HPF1
PARP1 + HPF1
+ARH3
wt
K9ac
K9me1
K9me2
K9me3
S10ph
ARH3
C
Histone
peptideADPr
Histone peptide
H4 wt
H4R3me2
H4K5ac
H4K8ac
H3 wt
H3K36ac
H3K36me1
H3K36 me2
H3K36me3

Figure S4. Canonical H3 histone marks reduce the efficiency of H3S10ADPr on H3 peptide. Related to Figure 3

(A) Autoradiogram showing PARP1/HPF1 ADPr signal on H3K9ac peptide following prior deacetylation treatments with HDAC2 and SIRT2. Coomassie staining of the SDS-PAGE is included.

(B) Autoradiogram showing ADPr, with or without subsequent addition of ARH3, with H3 (1-20aa) WT, K9ac, K9me1, K9me2, K9me3 and S10ph peptides. Coomassie staining of the SDS-PAGE is included.

(C) As in panel (B) except with H4 (1-23) WT, R3me2, K5ac, K8ac, H2A (1-17aa) WT, H3 (27-45aa) WT, K36ac, K36me1, K36me2, K36me3 peptides. Coomassie staining of the SDS-PAGE is included.

Figure S5

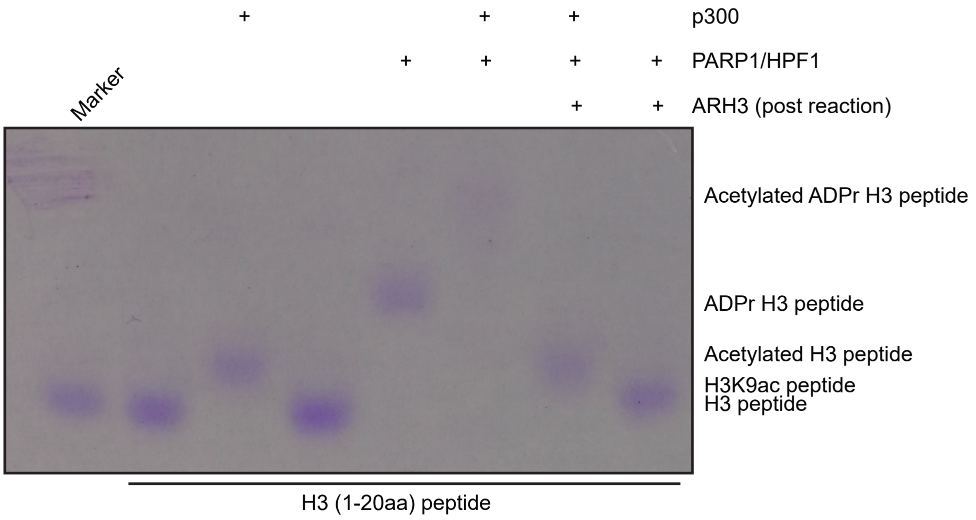

Figure S5. H3S10ADPr reduces the efficiency of subsequent H3K9 acetylation. Related to Figure 4 and 5

TBE gel of PARP1/HPF1 and/or p300 treatments of H3 (1-20aa) peptide. For details of the peptide separate technique please refer to Figure 5 and the related text. H3K9ac peptide was used as a marker. H3 peptides were ADP-ribosylated by PARP1/HPF1, prior to p300 acetylation treatment. One reaction (lane 7) was stopped after p300 incubation of Ser-ADP-ribosylated H3 peptide, then supplemented with ARH3 to remove ADPr (please note the mobility shift after the ARH3 treatment). ARH3 glycosylhydrolase removal of Ser-ADPr from non-acetylated H3 peptide is included as an additional control. Given the almost complete lack of H3K9ac detection by antibody in lane 6 of Figure 4A and a small but significant band shift in this figure (compare lane 7 to lanes 3 and 8), we note that Ser-ADPr H3 peptide can be acetylated by p300 at additional distal lysine sites in addition to K9.

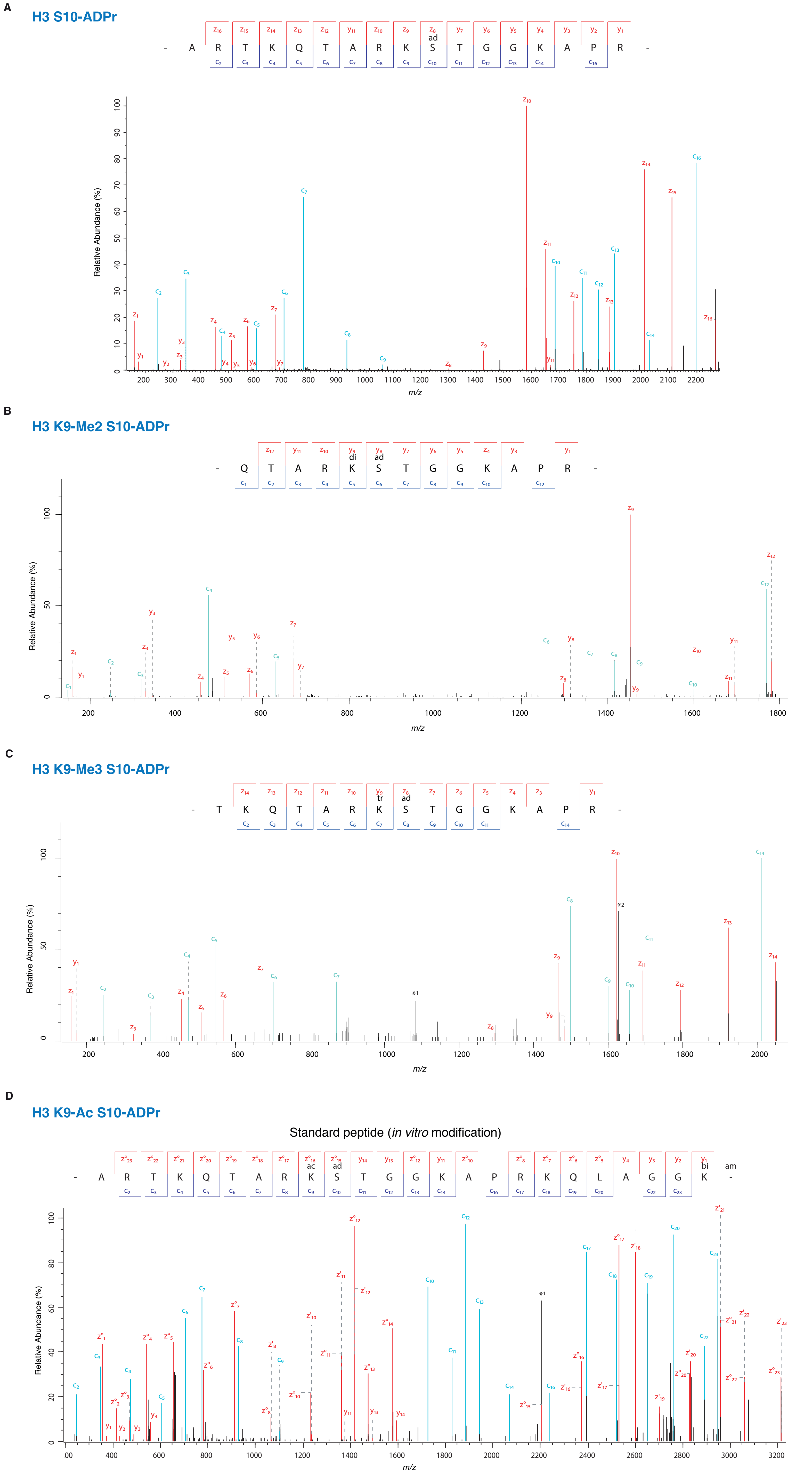
A
H3 S10-ADPr
- A R T K Q T A R K S T G G K A P R -
ad
Relative Abundance (%)
m/z
B
H3 K9-Me2 S10-ADPr
- Q T A R K S T G G K A P R -
di
ad
Relative Abundance (%)
m/z
C
H3 K9-Me3 S10-ADPr
- T K Q T A R K S T G G K A P R -
tr
ad
Relative Abundance (%)
m/z
D
H3 K9-Ac S10-ADPr
Standard peptide (in vitro modification)
- A R T K Q T A R K S T G G K A P R K Q L A G G K -
ac
ad
bi
am
Relative Abundance (%)
m/z

Figure S6. H3S10ADPr can coexist with mono-, di-, and tri-methylation of H3K9. Related to Figure 6

(A) High-resolution ETD fragmentation spectrum of a H3 peptide modified by ADP-ribose on serine 10 obtained from (Leidecker et al., 2016).

(B) High-resolution ETD fragmentation spectrum of a H3 peptide modified by di-methyl on lysine 9 and ADP-ribose on serine 10 obtained from (Leidecker et al., 2016).

(C) High-resolution ETD fragmentation spectrum of a H3 peptide modified by tri-methyl on lysine 9 and ADP-ribose on serine 10 obtained from (Leidecker et al., 2016). *[1] peak corresponding to an unfragmented co-eluting, co-isolated +2 precursor deconvoluted into the +1 state. *[2] peak corresponding to an unfragmented co-eluting, co-isolated +3 precursor deconvoluted into the +1 state.

(D) High-resolution ETD fragmentation spectrum of a H3-K9AcS10ad standard peptide (*in vitro* modification). *[1] peak corresponding to an unfragmented co-eluting, co-isolated +4 precursor deconvoluted into the +1 state.
